# A multiomics recovery factor predicts long COVID in the IMPACC study

**DOI:** 10.1172/JCI193698

**Published:** 2025-09-09

**Authors:** Gisela Gabernet, Jessica Maciuch, Jeremy P. Gygi, John F. Moore, Annmarie Hoch, Caitlin Syphurs, Tianyi Chu, Naresh Doni Jayavelu, David B. Corry, Farrah Kheradmand, Lindsey R. Baden, Rafick-Pierre Sekaly, Grace A. McComsey, Elias K. Haddad, Charles B. Cairns, Nadine Rouphael, Ana Fernandez-Sesma, Viviana Simon, Jordan P. Metcalf, Nelson I. Agudelo Higuita, Catherine L. Hough, William B. Messer, Mark M. Davis, Kari C. Nadeau, Bali Pulendran, Monica Kraft, Chris Bime, Elaine F. Reed, Joanna Schaenman, David J. Erle, Carolyn S. Calfee, Mark A. Atkinson, Scott C. Brakenridge, Esther Melamed, Albert C. Shaw, David A. Hafler, Alison D. Augustine, Patrice M. Becker, Al Ozonoff, Steven E. Bosinger, Walter Eckalbar, Holden T. Maecker, Seunghee Kim-Schulze, Hanno Steen, Florian Krammer, Kerstin Westendorf, IMPACC Network, Bjoern Peters, Slim Fourati, Matthew C. Altman, Ofer Levy, Kinga K. Smolen, Ruth R. Montgomery, Joann Diray-Arce, Steven H. Kleinstein, Leying Guan, Lauren I.R. Ehrlich

**Affiliations:** 1 Yale School of Medicine, New Haven, Connecticut, USA.; 2 Northwestern Feinberg School of Medicine, Chicago, Illinois, USA.; 3 The University of Texas at Austin, Austin, Texas, USA.; 4 Clinical and Data Coordinating Center (CDCC), Precision Vaccines Program, Boston Children’s Hospital, Harvard Medical School, Boston, Massachusetts, USA.; 5 Benaroya Research Institute, University of Washington, Seattle, Washington, USA.; 6 Baylor College of Medicine and the Center for Translational Research on Inflammatory Diseases, Houston, Texas, USA.; 7 Brigham and Women’s Hospital, Harvard Medical School, Boston, Massachusetts, USA.; 8 Case Western Reserve University and University Hospitals of Cleveland, Cleveland, Ohio, USA.; 9 Drexel University, Tower Health Hospital, Philadelphia, Pennsylvania, USA.; 10 Emory School of Medicine, Atlanta, Georgia, USA.; 11 Icahn School of Medicine at Mount Sinai, New York, New York, USA.; 12 Oklahoma University Health Sciences Center, Oklahoma City, Oklahoma, USA.; 13 Oregon Healthand Science University, Portland, Oregon, USA.; 14 Stanford University School of Medicine, Palo Alto, California, USA.; 15 University of Arizona, Tucson, Arizona, USA.; 16 David Geffen School of Medicine at the UCLA, Los Angeles California, USA.; 17 UCSF, San Francisco, California, USA.; 18 University of Florida, Gainesville, Florida, USA.; 19 National Institute of Allergy and Infectious Diseases (NIAID), NIH, Bethesda, Maryland, USA.; 20 Precision Vaccines Program, Boston Children’s Hospital, Harvard Medical School, Boston, Maryland, USA.; 21 La Jolla Institute for Immunology, La Jolla, California, USA.; 22 The Immunophenotyping Assessment in a COVID-19 Cohort (IMPACC) Network members (detailed in Supplemental Acknowledgments).

**Keywords:** Immunology, Infectious disease, Biomarkers, COVID-19, Machine learning

## Abstract

**BACKGROUND:**

Following SARS-CoV-2 infection, approximately 10%–35% of patients with COVID-19 experience long COVID (LC), in which debilitating symptoms persist for at least 3 months. Elucidating the biologic underpinnings of LC could identify therapeutic opportunities.

**METHODS:**

We utilized machine learning methods on biologic analytes provided over 12 months after hospital discharge from more than 500 patients with COVID-19 in the IMPACC cohort to identify a multiomics “recovery factor,” trained on patient-reported physical function survey scores. Immune profiling data included PBMC transcriptomics, serum O-link and plasma proteomics, plasma metabolomics, and blood mass cytometry by time of flight (CyTOF) protein levels. Recovery factor scores were tested for association with LC, disease severity, clinical parameters, and immune subset frequencies. Enrichment analyses identified biologic pathways associated with recovery factor scores.

**RESULTS:**

Participants with LC had lower recovery factor scores compared with recovered participants. Recovery factor scores predicted LC as early as hospital admission, irrespective of acute COVID-19 severity. Biologic characterization revealed increased inflammatory mediators, elevated signatures of heme metabolism, and decreased androgenic steroids as predictive and ongoing biomarkers of LC. Lower recovery factor scores were associated with reduced lymphocyte and increased myeloid cell frequencies. The observed signatures are consistent with persistent inflammation driving anemia and stress erythropoiesis as major biologic underpinnings of LC.

**CONCLUSION:**

The multiomics recovery factor identifies patients at risk of LC early after SARS-CoV-2 infection and reveals LC biomarkers and potential treatment targets.

**TRIAL REGISTRATION:**

ClinicalTrials.gov NCT04378777.

**FUNDING:**

National Institute of Allergy and Infectious Diseases (NIAID), NIH (3U01AI167892-03S2, 3U01AI167892-01S2, 5R01AI135803-03, 5U19AI118608-04, 5U19AI128910-04, 4U19AI090023-11, 4U19AI118610-06, R01AI145835-01A1S1, 5U19AI062629-17, 5U19AI057229-17, 5U19AI057229-18, 5U19AI125357-05, 5U19AI128913-03, 3U19AI077439-13, 5U54AI142766-03, 5R01AI104870-07S1, 3U19AI089992-09, 3U19AI128913-03, and 5T32DA018926-1, 3U19AI1289130, U19AI128913-04S1, R01AI122220); NIH (UM1TR004528); and National Science Foundation (NSF) (DMS2310836).

## Abstract 2

Identification of a multi-omics factor, encompassing interpretable biological features, that both underlies Long COVID (LC) symptoms and predicts LC occurrence at hospital admission in the IMPACC cohort.

## Graphical abstract

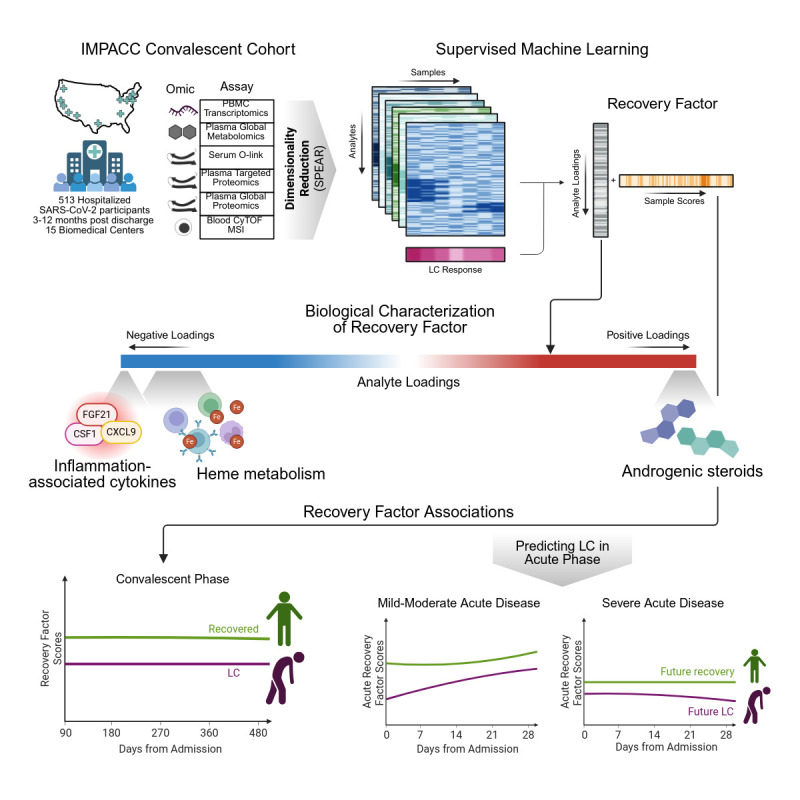

## Introduction

Long COVID (LC) has become a pressing public health concern, affecting approximately 10%–35% of surviving individuals infected with SARS-CoV-2, or 15–20 million people in the United States and more than 60 million people worldwide (1, 2). In July 2024, the National Academies of Sciences, Engineering, and Medicine (NASEM) released an updated definition of LC, characterizing it as a chronic condition arising after SARS-CoV-2 infection that persists for at least 3 months, irrespective of acute disease severity (2). LC can encompass a wide range of physical and cognitive symptoms and can lead to new or worsening neurological, psychiatric, cardiovascular, pulmonary, endocrine, and gastrointestinal conditions, among others (1–12).

Several studies have identified demographic and clinical risk factors for LC (13–15), including older age (16–18), female sex (16–21), and longer hospital stays (18, 22). Higher viral loads (18, 19) and lower anti–SARS-CoV-2 antibody titers (18, 23, 24) during the acute infection phase have also been associated with LC development. Several nonmutually exclusive hypotheses may explain the etiology of LC, including persistent viral infection (25, 26), chronic inflammation (26–29), latent herpesvirus reactivation (26, 30, 31), immune dysregulation (29, 32, 33), complement dysregulation (34), and autoimmunity (35, 36). Despite substantial efforts, no consensus exists on the mechanisms of LC pathogenesis, and validation of the molecular findings across cohorts has been challenging. Additionally, most existing studies rely on measurements from a single or limited number of assays and are confined to restricted sampling time points during the acute or convalescent phases of the disease. Accordingly, a large-scale multiomics longitudinal study spanning both acute infection and convalescent disease phases could help elucidate the molecular mechanisms underlying LC.

The Immunophenotyping Assessment in a COVID-19 Cohort (IMPACC) study (37) offers a unique opportunity to investigate the temporal dynamics of multiomics immune profiles during the acute and convalescent COVID-19 infection phases in a clinically well-characterized cohort of hospitalized patients from across the United States. Data from the IMPACC study can be leveraged to identify molecular correlates of post–acute symptom development or resolution for 1 year after hospital discharge. This cohort has previously been studied to characterize multiomics determinants of acute COVID-19 severity and mortality (38–40). Another study of the IMPACC cohort identified LC study participants who reported outcome deficits up to 12 months after COVID-19 hospital discharge (18). Clinical characteristics such as female sex or a higher respiratory SARS-CoV-2 viral burden with lower antibody titers against the SARS-CoV-2 spike protein during acute disease were associated with persistent deficits after hospital discharge. B cell lymphopenia and elevated FGF21 during the acute phase were also characteristics of participants who developed LC (18). However, longitudinal immune profiles of IMPACC participants experiencing LC during the convalescent phase have not yet been compared with those of IMPACC participants who experienced minimal deficits during convalescence, an analysis that could reveal LC biomarkers and uncover biological processes underlying the disease.

In the current work, we applied supervised multiomics integration methods to develop interpretable models that differentiate participants with LC from recovered individuals on the basis of their longitudinal immunophenotyping profiles during the convalescent disease phase. We identified key biological programs and biomarkers driving LC classification. Our findings highlight persistent inflammation, elevated heme metabolism associated with anemia, and reduced plasma concentrations of androgenic steroids as characterizing features of LC, independent of acute disease severity or SARS-CoV-2 vaccination status after hospital discharge. Notably, these molecular profiles were already detectable during acute disease, suggesting their potential value as early predictive biomarkers for identifying patients at risk of developing LC. Additionally, despite a general lack of consensus about the definition of LC or consistency in the timing of sampling across different studies, we validated dysregulation of the heme metabolism signature in 2 independent LC cohorts that included nonhospitalized patients with COVID-19. These findings provide valuable insights into the molecular underpinnings of LC and offer a foundation for future research aimed at improving diagnostics and developing targeted interventions.

## Results

### Longitudinal multiomics profiling of LC.

**Figure 1:**
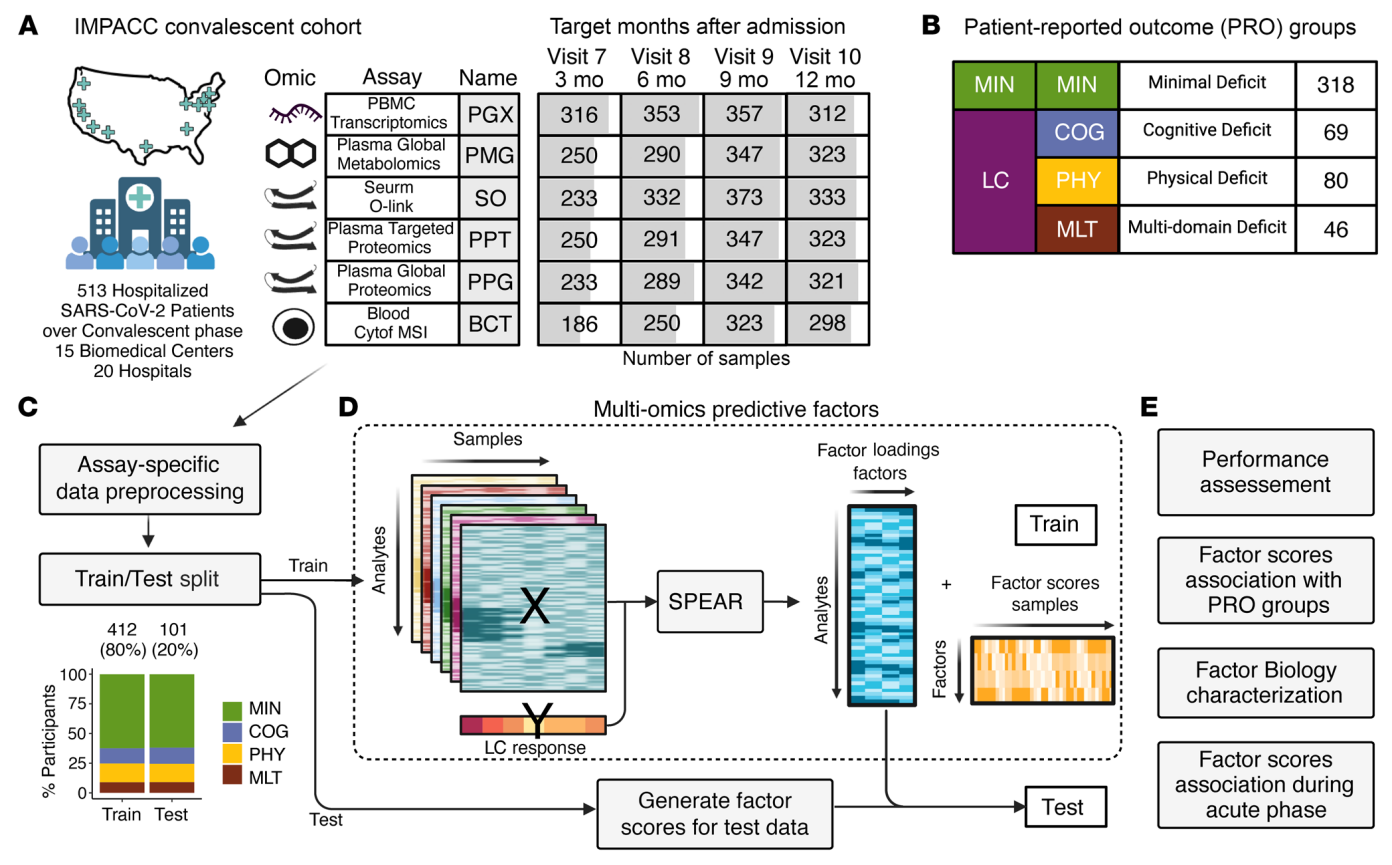
Multiomics data overview and generation of a predictive LC factor. (**A**) Number of samples used in the multiomics data integration strategy by assay (rows) and scheduled time of collection (columns). Shading indicates the frequency of samples with data availability at the indicated visit. (**B**) Patient classification in PRO clusters according to the PRO survey scores (18). (**C**) Individual assay data were preprocessed and split into train and test cohorts by participant in an 80/20 split, maintaining the proportion of PRO cluster participants in each partition. (**D**) Preprocessed assay data and LC response outcomes for the train cohort were used to identify multiomics predictive factors with SPEAR. Factor scores were then calculated for the test cohort. (**E**) The performance of the multiomics predictive factors to classify patients according to the presence or absence of LC was assessed on the train cohort via cross-validation and then validated on the test cohort. The predictive factor scores were confirmed to be associated with LC after correcting for possible confounding variables. In-depth analysis of enriched biological pathways and significant analytes relevant for the prediction was performed. Factor scores were computed for the acute infection immune profiles, and association analysis with LC at these early time points was performed. See also Supplemental Figures 1 and 2.

The IMPACC study included 1,164 participants admitted to 20 US hospitals for SARS-CoV-2 infection between May 2020 and March 2021 (37). Clinical data collection and immunophenotyping were performed longitudinally during the acute disease phase within 72 hours of hospital admission and 4, 7, 14, 21, and 28 days after hospital admission (visits 1–6, respectively). Surviving participants were contacted 3, 6, 9, and 12 months after hospital discharge (visits 7–10, respectively) to complete patient-reported outcome (PRO) and symptom surveys during the convalescent phase and to provide biosamples for immunophenotyping assays. Of the 702 participants who could be reached by the study team after discharge, 513 were included in the IMPACC convalescent cohort (Supplemental Figure 1; supplemental material available online with this article; https://doi.org/10.1172/JCI193698DS1). These participants were selected because they survived at least 28 days of hospitalization, completed at least 1 PRO survey, and provided at least 1 biosample during the convalescent period (Figure 1A and Supplemental Table 1) (18). IMPACC core laboratories performed immunophenotyping in both the acute and convalescent phases, including measurements of inflammatory mediators in blood serum via Olink (SO), global blood plasma metabolomics (PMG), global and targeted blood plasma proteomics (PPG and PPT), PBMC transcriptomics (PGX), whole blood cell frequencies measured with mass cytometry by time of flight (CyTOF), and CyTOF mean marker signal intensity measurements (BCT).

LC status was defined in this cohort according to the participant’s response to post-discharge surveys that captured symptoms and PRO measures evaluating general health and deficits in specific domains. Participants who responded to at least 1 set of post-discharge surveys were assigned to PRO clusters according to latent class modeling and clustering using standardized scores of the PRO survey measures (18) (PRO survey score details can be found in the Supplemental Methods). PRO clusters were classified as participant clusters with no or minimal deficits (MIN), or with deficits attributed to LC in several domains: physical predominant (PHY), mental/cognitive predominant (COG), and multi/pan domain (MLT) (18) (Figure 1B).

In this study, we utilized multiomics immunophenotyping profiles from participant biosamples obtained during the convalescent disease phase to develop interpretable models for predicting LC and exploring the underlying molecular mechanisms. To assess model performance, we split the convalescent cohort into an 80% train cohort and a 20% test cohort, maintaining the proportions of participants in each PRO cluster (Figure 1C), with no noticeable imbalance in other clinical characteristics or biosample availability between the cohorts (Supplemental Figure 2). We then used Signature-based multiPle-omics intEgration via lAtent factoRs (SPEAR) (41), a supervised Bayesian factor model for the identification of multiomics features, to integrate the high-dimensional data and construct multiomics predictive factors from immune profiles obtained during the convalescent phase in the train cohort. We assessed their predictive performance by repeated cross-validation on the train cohort and validated the performance of the selected model on the test cohort (Figure 1, D and E). To identify immune programs captured in the predictive factors, we conducted in-depth analyses of enriched biological pathways and analytes identified as highly relevant for the model’s predictive performance and performed associations with assay data not included in model training, such as blood CyTOF cell frequencies.

### Multiomics factors are predictive of LC.

**Figure 2:**
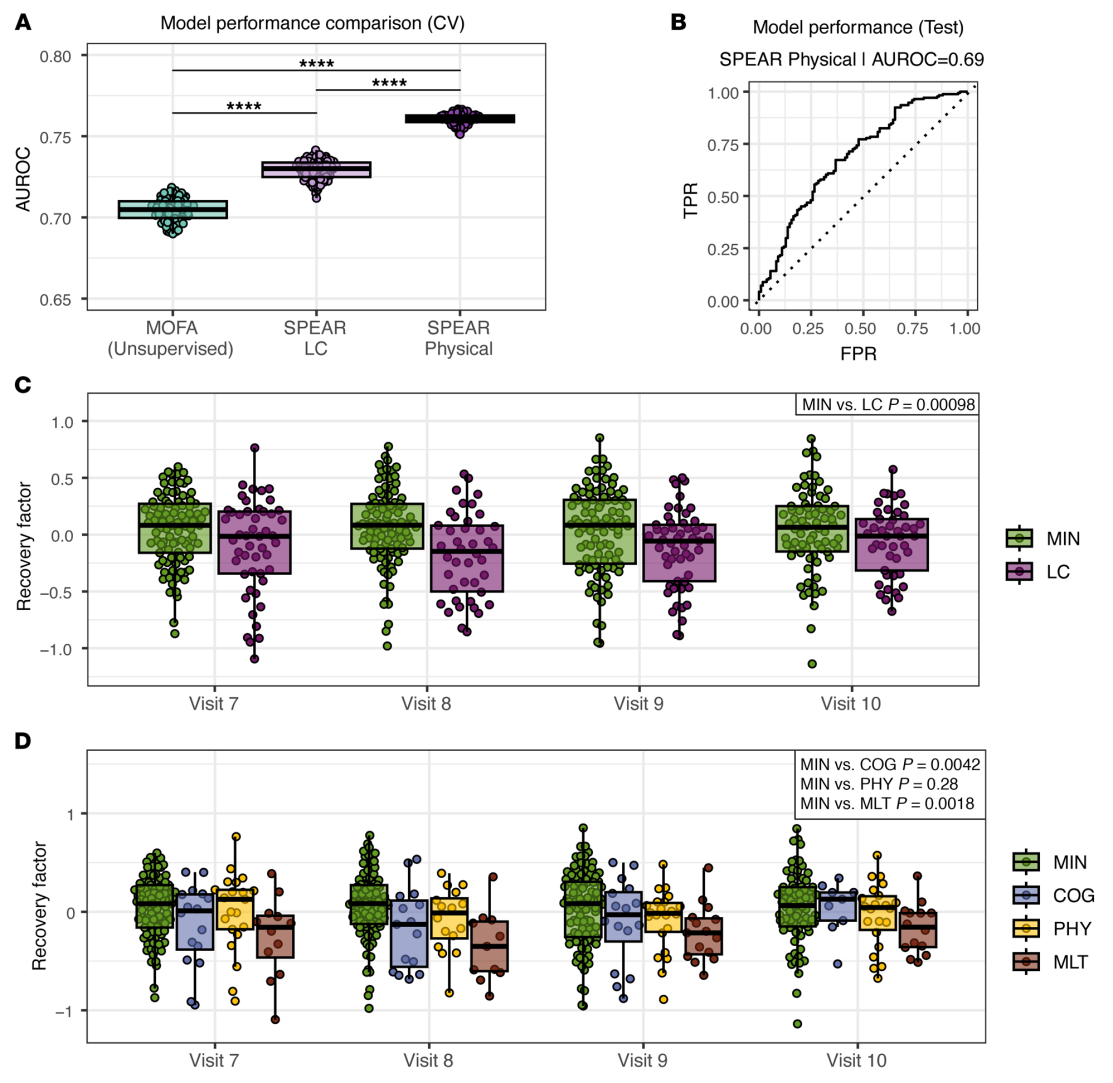
Identification of a convalescent multiomics recovery factor that discriminates LC. (**A**) Predictive performance of a lasso model trained on the MOFA and SPEAR factors to discriminate LC versus MIN at the event level. The mean AUROC of a 10-fold cross-validation on the train cohort for 100 bootstrapped model training repetitions is shown. Significance was calculated by standard normal approximation of bootstrapped differences between models (*t* test, adj. ****P ≤ 0.0001). CV, cross validation. (**B**) Predictive performance of the SPEAR Physical model to discriminate LC versus MIN on the test cohort. The ROC curve of model (solid line), random classifier (dashed line), and AUROC value are shown. TPR, true-positive rate; FPR, false-positive rate. (**C**) Recovery factor scores for the test cohort of the MIN and LC groups at 3 months (visit 7), 6 months (visit 8), 9 months (visit 9), and 12 months (visit 10) after hospital discharge. (**D**) Recovery factor scores of the individual PRO clusters by visit for the test cohort. *P* values in **C** and **D** show the significance of the recovery factor score association with MIN versus LC and pairwise PRO cluster combinations using a goodness-of-fit χ^2^ test. See also Supplemental Figure 3–5.

We focused on predicting LC in the convalescent cohort using multiomics immune profiling data collected during the convalescent phase. We constructed several SPEAR models to generate supervised factors. These models used either PRO survey scores from PRO Measurement Information System (PROMIS) surveys (SPEAR Physical, SPEAR Cognitive, SPEAR Mental, SPEAR Impact, SPEAR Dyspnea) or the binary LC labels assigned to each participant (SPEAR LC) as response variables (Supplemental Figure 3A). We trained models on these different response variables, since binary LC labels (presence or absence of LC) per participant could omit valuable information captured by numeric PRO survey scores at each visit. Note that PRO survey scores were available for participants in all LC clinical PRO clusters, so each individual SPEAR model (e.g., SPEAR Physical) was trained using data from participants in all 4 PRO clusters (MIN, PHY, COG, MLT) (18). The SPEAR Physical model performed best among all models trained on PRO survey scores (Supplemental Figure 3B) and outperformed the model trained on binary LC labels (Figure 2A). Additionally, all SPEAR models, obtained with the Multiomics Factor Analysis (MOFA) framework (42), outperformed equivalent models trained on unsupervised multiomics factors, which do not consider a response variable during the factor construction step (Figure 2A and Supplemental Figure 3B). The SPEAR Physical model achieved an area under the receiver operating characteristic curve (AUROC) of 0.69 for predicting LC presence or absence in the test cohort (Figure 2B). The SPEAR Physical Factor, learned by the SPEAR physical model, was significantly associated with LC in the test cohort after correcting for sex and age (*P* = 0.00098, effect size 0.44), two variables previously associated with LC in our cohort (18) (Figure 2C). Sparse lasso regression models to reconstruct SPEAR Physical factor scores utilizing all analytes included in the model or analytes from individual assays showed that the model that included all analytes best reconstructed the factor scores, indicating that factor scores captured contributions from multiple omics (Supplemental Figure 3C). The SPEAR Physical Factor scores were significantly higher for participants in the MIN group compared with scores for the LC group, so we termed this factor the “recovery factor” (Figure 2C, Supplemental Figure 4, and Supplemental Figure 5). Recovery factor scores were significantly associated with PRO clusters (*P* = 0.0009); however, they showed a differential ability to identify individual LC deficit domains, with significant differences between MIN and COG (*P* = 0.0042, effect size 0.61) and MIN and MLT (*P* = 0.0018, effect size 0.60) PRO clusters, but not MIN and PHY clusters (*P* = 0.28, effect size 0.47) (Figure 2D and Supplemental Figure 5B). Taken together, the recovery factor is a multiomics model composed of biologic analyte levels during the convalescent phase of COVID-19 that distinguished MIN from LC over 12 months after hospital discharge in the IMPACC cohort.

### Functional characterization of the recovery factor.

**Figure 3:**
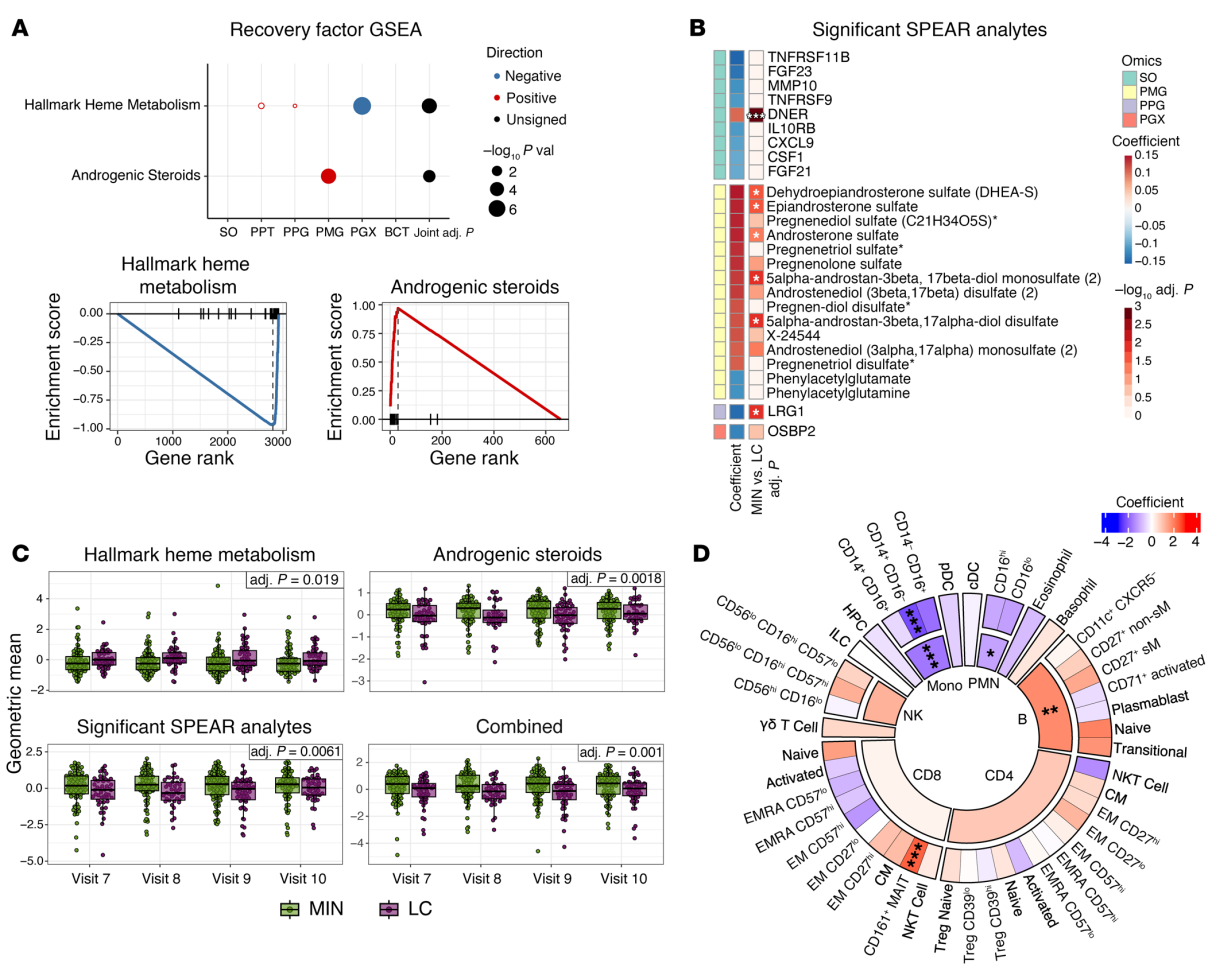
Heme metabolism and androgenic steroid pathways, inflammation-associated serum factors, and altered immune cell composition are associated with the recovery factor during convalescence. (**A**) GSEA identified heme metabolism and androgenic steroid pathways as being significantly associated with the recovery factor, with significance shown per assay, as well as across assays (joint adj. *P* < 0.05). (**B**) Twenty-six significant analytes (SPEAR Bayesian posterior selection probability ≥0.95) in the recovery factor across different assays (left) were identified using SPEAR factor loadings (middle; coefficient in the factor), and each was tested for association in the test cohort with MIN versus LC groups (right; adj. intercept *P* value). (**C**) Geometric means of analytes from the significantly enriched gene and metabolite sets and/or significant SPEAR analytes are shown per sample at each convalescent visit in the test cohort. The combined geometric mean score includes leading edge analytes from the hallmark heme metabolism and androgenic steroids pathways, and the significant SPEAR analytes. The *P* values indicate significance of the association with MIN versus LC. (**D**) Association in the full cohort of whole blood cell counts determined by CyTOF with the recovery factor for parent and child immune cell types. Mono, monocytes; B, B lymphocytes; CD4, CD4^+^ T lymphocytes; CD8, CD8^+^ T lymphocytes; CD27^+^ non-sM, CD27^+^ nonswitched memory; CD27^+^ sM, CD27^+^ switched memory. For a full list of the child populations, see Supplemental Table 3. (adj. *P < 0.05, adj. **P < 0.01, adj. ***P < 0.001). See also Supplemental Figure 6.

To characterize the biologic processes underlying the recovery factor, we performed gene set enrichment analysis (GSEA) for each of the multiomics assays based on the SPEAR model’s internal ranking of the relative importance of each feature for predicting the PRO PROMIS Physical score. The hallmark heme metabolism transcriptomic pathway was negatively associated with the recovery factor, indicating upregulation in participants with LC, whereas the androgenic steroids metabolite set was positively associated with the recovery factor, indicating downregulation in participants with LC (Figure 3A). Evaluated individually, several leading-edge analytes in the hallmark heme metabolism gene set and androgenic steroids subpathway metabolite set showed significant associations with LC status (Supplemental Figure 6, A and B).

SPEAR performs internal significance testing to determine the importance of each analyte in predicting the response variable. The SPEAR Physical model identified 26 analytes across 4 assays as significant in the recovery factor (SPEAR Bayesian posterior selection probability ≥0.95), and we assessed the associations of these features with LC status in the test cohort, adjusting for age and sex (Figure 3B and Supplemental Figure 6E). Nine of these 26 analytes were from the serum Olink assay. Of these, DNER, a noncanonical Notch ligand implicated in promoting tumor growth, metastasis, and wound healing (43, 44), was significantly reduced in LC participants, consistent with a prior study of plasma proteins in LC (28). The remaining serum Olink analytes were negatively associated with the recovery factor. These included proteins and cytokines associated with chronic inflammatory conditions (45–50), particularly endothelial/vascular inflammation (FGF23, FGF21, CXCL9, TNFRSF11B, and TNFRSF9 [CD137]), as well as inflammation-associated myeloid regulators (51–53) (MMP10 and CSF1). Elevated IL10RB levels have been associated with worse outcomes in acute SARS-CoV-2 infection (54), consistent with elevation under inflammatory conditions. Leucine-rich alpha-2 glycoprotein 1 (LRG1), a protein elevated in participants with LC, is induced by IL-6 and other inflammatory cytokines and has been implicated in angiopathic activity (55–57). Phenylacetylglutamate and phenylacetylglutamine are gut microbiota–derived metabolites associated with vascular inflammation and thrombosis (58). Finally, the OSBP2 transcript, which encodes an oxysterol binding protein (59), was also identified as a leading edge gene in the Hallmark Heme Metabolism gene set elevated in LC participants.

Several metabolites from the androgenic steroids pathway were represented in the 26 significant analytes and were positively associated with the recovery factor, indicating that higher levels correlate with better physical function. When these metabolites were tested individually for association with LC status, five [DHEA-S, epiandrosterone sulfate, androsterone sulfate, 5α-androstan-3β,17β-diol monosulfate (2), 5α-androstan-3β,17α-diol disulfate] were significantly lower in LC participants, adjusting for age and sex (Figure 3B). Androgens can suppress inflammation (60), suggesting that higher levels of androgenic steroids in MIN participants could reflect better control of chronic inflammation. These findings are consistent with reports showing lower sex hormone levels in individuals with LC (31). Five metabolites related to pregnenolone were also represented in the significant SPEAR analytes (Figure 3B). Pregnenolone is synthesized from cholesterol as the first step of the steroid hormone biosynthesis pathway and is known to have potent effects as an inhibitor of inflammation (61) and as a neurosteroid (62). Altogether, these findings are consistent with a prominent role for persistent inflammation in LC with dysregulation of key analytes that may contribute to LC symptoms, including those that drive angiopathy, reduce wound healing, and alter heme metabolism.

The feature sets from heme metabolism and androgenic steroids identified by GSEA, combined with the significant SPEAR analytes, represent 73 unique features that potentially condense the predictive power of the recovery factor into a smaller feature set. To test this hypothesis, we calculated the geometric mean of the 43 leading-edge heme metabolism and 12 androgenic steroid features, as well as the 26 significant SPEAR analytes. All 3 geometric mean scores were significantly associated with LC in the test cohort (Figure 3C). Furthermore, the combined score, including analytes from all 3 feature sets, discriminates MIN and LC participants with even greater significance (Figure 3C). Thus, while the recovery factor consists of weighted contributions from 6,807 features, we have identified a smaller set of 73 unique features that discriminates participants according to LC status in the convalescent period.

Consistent with our finding, studies involving 2 separate cohorts have reported upregulation of the hallmark heme metabolism pathway in individuals with LC. In Hanson et al. (29), hospitalized and nonhospitalized participants with persisting symptoms 1–3 months after acute SARS-CoV-2 infection had higher hallmark heme metabolism signatures than did participants without persisting symptoms. In the study by Karisola et al. (63), which included only nonhospitalized patients with COVID-19, men with persisting symptoms 3 months after acute SARS-CoV-2 infection had higher hallmark heme metabolism signatures than did men without persisting symptoms. To determine whether the same heme metabolism–related genes were dysregulated in participants with LC in the IMPACC and external cohorts, we used the leading-edge genes from the significant hallmark heme metabolism pathway in our GSEA results (Supplemental Figure 6A) and calculated the geometric mean scores in whole blood transcriptomics profiles from Hanson et al. (29) and PBMC transcriptomics profiles from Karisola et al. (63). These heme metabolism leading-edge gene scores significantly differentiated participants with persistent symptoms from those with resolved symptoms in both cohorts, including both sexes (Supplemental Figure 6, C and D). The generalization of elevated expression of this heme metabolism gene set in nonhospitalized and hospitalized patients with COVID-19 who experienced LC in 3 independent and varied cohorts underscores its centrality for LC pathology.

Prior studies have identified altered leukocyte frequencies as a feature of LC (18, 26, 29, 31, 33). To determine whether similar cellular changes were associated with the recovery factor, we analyzed whole blood CyTOF cell frequencies for 15 parent and 46 child immune cell types in our cohort during convalescence (Figure 3D). Several cell subsets were significantly associated with the recovery factor. B cells and CD161^+^ muc–osal-associated invariant T (MAIT) cells were positively associated with the recovery factor. In contrast, polymorphonuclear neutrophils (PMNs) and monocytes, specifically the CD14^+^CD16^–^ classical monocyte subset, were negatively associated with the recovery factor. Together, these findings suggest that elevated monocytes and neutrophils, along with decreased B cells, are associated with prolonged inflammation during LC. These findings are consistent with a previous report that monocytes are elevated in men with LC (31). The decrease in MAIT cells could be another effect of sustained inflammation, as reduced circulation of MAIT cells has been associated with chronic HIV (64) and hepatitis C (65) infections.

### The recovery factor is associated with clinical characteristics and multiple PROs in the convalescent period.

**Figure 4:**
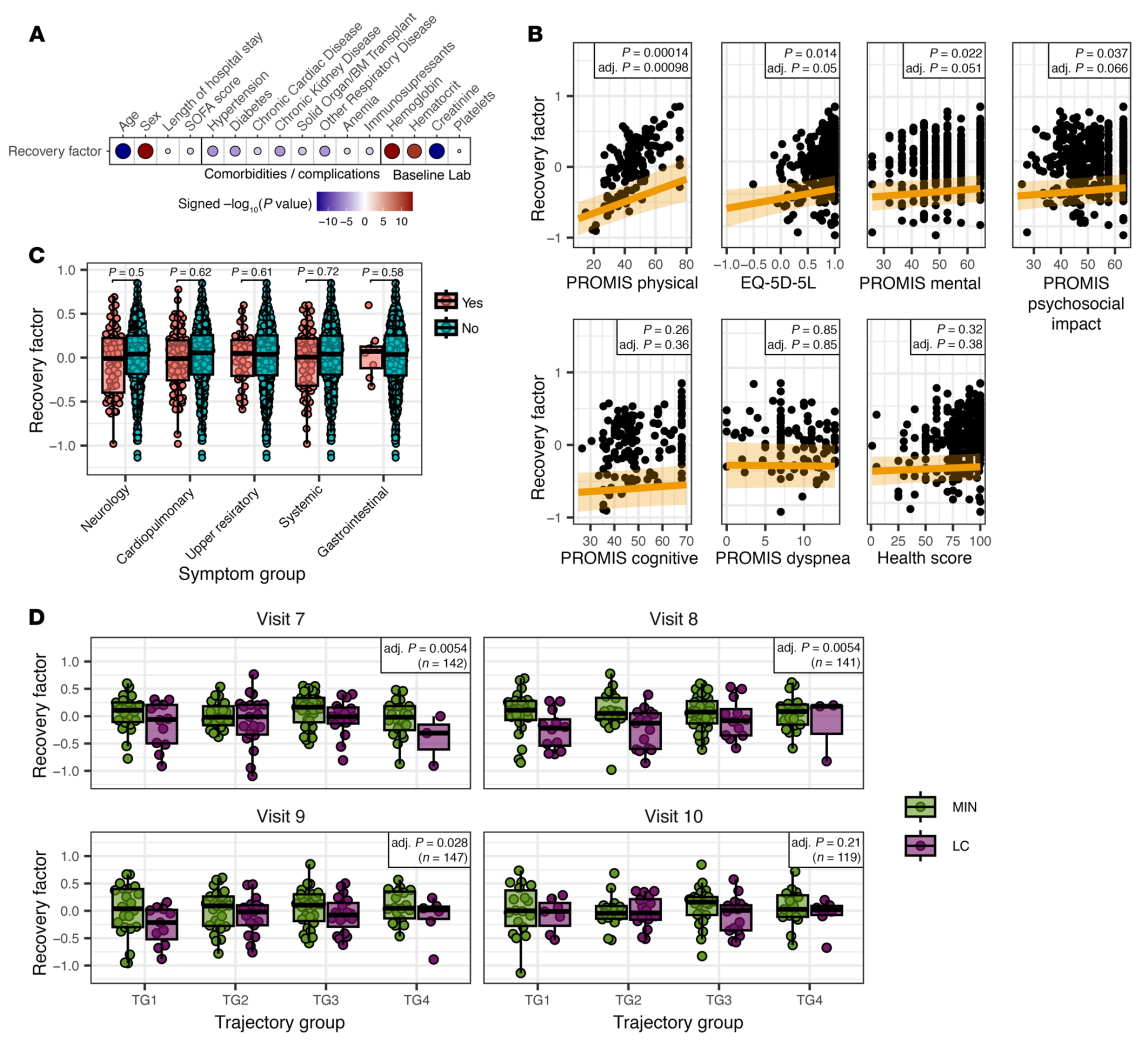
Associations of clinical measurements with recovery factor scores. (**A**) Association of recovery factor scores with clinical features (demographics, comorbidities, complications, and baseline laboratory measurements). Dot plot shows the signed adjusted *P* values indicating the clinical feature term significance from a linear mixed-effects model, with enrollment site and participant as random effects to explain the convalescent phase recovery factor scores. Sex and discretized age were further adjusted as fixed effects for clinical features other than sex and age. Only significant associations (adj. *P* < 0.05) are shown. (**B**) Associations of recovery factor scores with individual PRO survey scores (PROMIS scale scores, EQ-5D-5L and health score) in the test cohort. Raw and adjusted *P* values indicate the PRO score term significance in linear mixed-effects models. (**C**) Associations of recovery factor scores with each indicated symptom group in the test cohort: neurological, cardiopulmonary, upper respiratory, systemic, gastrointestinal. Numbers are the uncorrected significance (*P* values) of the symptom group term in linear mixed-effects models. (**D**) Recovery factor scores per participant in the test cohort, separated into MIN and LC groups by acute phase trajectory groups, stratified by visit. *P* values for **B**–**D** show the endpoint term of a linear mixed-effects model with sex, discretized admission age, and trajectory group as fixed effects and enrollment site as a random effect. No individual MIN versus LC comparisons were significant after *P* value correction.

We next evaluated whether the recovery factor was associated with clinical features and additional clinical outcomes. We tested the association of recovery factor scores with clinical features at hospital admission (visit 1), including demographics, comorbidities, complications, and baseline laboratory measurements (Figure 4A). Several demographic and clinical measurements were significantly associated with recovery factor scores, including age, sex, length of hospital stay, and the Sequential Organ Failure Assessment (SOFA) score. Notably, anemia at hospital discharge was negatively associated with the recovery factor, whereas hemoglobin (Hgb) and hematocrit baseline measurements showed a significant positive association (Figure 4A). We additionally conducted association testing with PRO scores from surveys conducted during the same visit at which the recovery score was assessed in participants across the convalescent period, correcting for age and sex. The recovery factor score was significantly associated in the test cohort with the PROMIS Physical score, on which the model was trained (Figure 4B), and the EQ-5D-5L score, both of which contained questions assessing physical function (Figure 4B). The recovery factor also correlated with PROMIS Mental and Psychosocial Impact scores, although these associations were not significant after *P*-value correction (Figure 4B). We also tested whether recovery factor scores associated with reported clinical symptoms in the 7 days prior to each visit but found no significant association (Figure 4C).

There is a lack of consensus about whether LC is associated with the severity of acute disease. A previous analysis of clinical features from the IMPACC cohort showed no association between acute infection severity, as assessed by clinical trajectory groups, and LC development (18). However, other studies have found an association (6). Thus, we sought to determine whether acute disease severity contributed to the association between recovery factor scores and LC status in our cohort. Clinical severity in the IMPACC cohort during the acute phase was defined by unsupervised clustering of the respiratory ordinal score over time, taking discharge status and limitations into account, with trajectory group 1 (TG1) representing the mildest and TG4 the most severe disease course among participants who survived for at least 28 days after hospitalization (66). After correcting for acute phase trajectory group assignment, recovery factor scores remained significantly associated with LC at the first 3 convalescent time points (Figure 4D), indicating that acute clinical severity does not contribute to the association between participant recovery factor scores in the convalescent disease phase and LC status.

### Sex affects recovery factor scores.

LC occurs more frequently in women than men, despite a higher percentage of men with severe acute COVID-19 disease (21, 67). In the IMPACC convalescent cohort, nearly half of the female participants presented with long-term deficits compared with only approximately 30% of male participants (Supplemental Figure 7A). Assignment to clinical LC subtypes was not influenced by sex, with similar proportions and numbers of male and female LC participants assigned to the COG, PHY, and MLT PRO clusters (Supplemental Figure 7A). However, consistent with the known influence of sex on LC status, sex was a statistically significant covariate in the recovery factor association with LC status from Figure 2C (*P* = 3.6 × 10^–7^) and with PRO clusters from Figure 2D (adjusted [adj.] *P* < 0.001 in all pairwise comparisons). Thus, we tested whether the recovery factor discriminates LC in both men and women by repeating our associations with LC status in the test cohort separated by sex. Recovery factor scores were significantly associated with the binary assignment to LC versus MIN groups in women but not in men after *P* value adjustment (Supplemental Figure 7B), although the trend of lower scores in participants with LC persisted in men. When considering individual PRO groups, recovery factor scores discriminated between MIN versus COG and MIN versus MLT groups for women and MIN versus MLT PRO groups for men (Supplemental Figure 7C). Given that the LC incidence is lower in men, it is notable that recovery factor scores were generally higher for men than for women, regardless of LC status. Geometric mean scores of the leading-edge analytes in the recovery factor from the heme metabolism and androgenic steroid pathways and the significant SPEAR analytes lost significance in 1 or both sexes when the cohort was divided into men and women (Supplemental Figure 7D). Notably, though, their combined score remained significantly associated with LC in both sexes (Supplemental Figure 7D).

### Vaccination is not associated with altered recovery factor scores.

Our cohort was enrolled prior to the national SARS-CoV-2 vaccine rollout for the general population. During the longitudinal post-hospitalization follow-up period, as vaccines became broadly available, close to 75% of the participants in the IMPACC convalescent cohort received a SARS-CoV-2 vaccine (Supplemental Figure 8, A and B). To assess the potential influence of the vaccine response on the immune profiling data and thus the recovery factor, we compared recovery factor scores per visit for events occurring before and after the first vaccine dose, as well as events occurring within a 3-week period after any vaccine dose, when vaccine responses have been shown to affect immune profiles (68, 69). No significant difference was found in recovery factor scores across these comparisons, indicating a negligible effect of vaccination on immune profiles related to LC in our patient cohort (Supplemental Figure 8, C and D).

### Recovery factor scores during the acute disease phase associate with LC status during convalescence.

**Figure 5:**
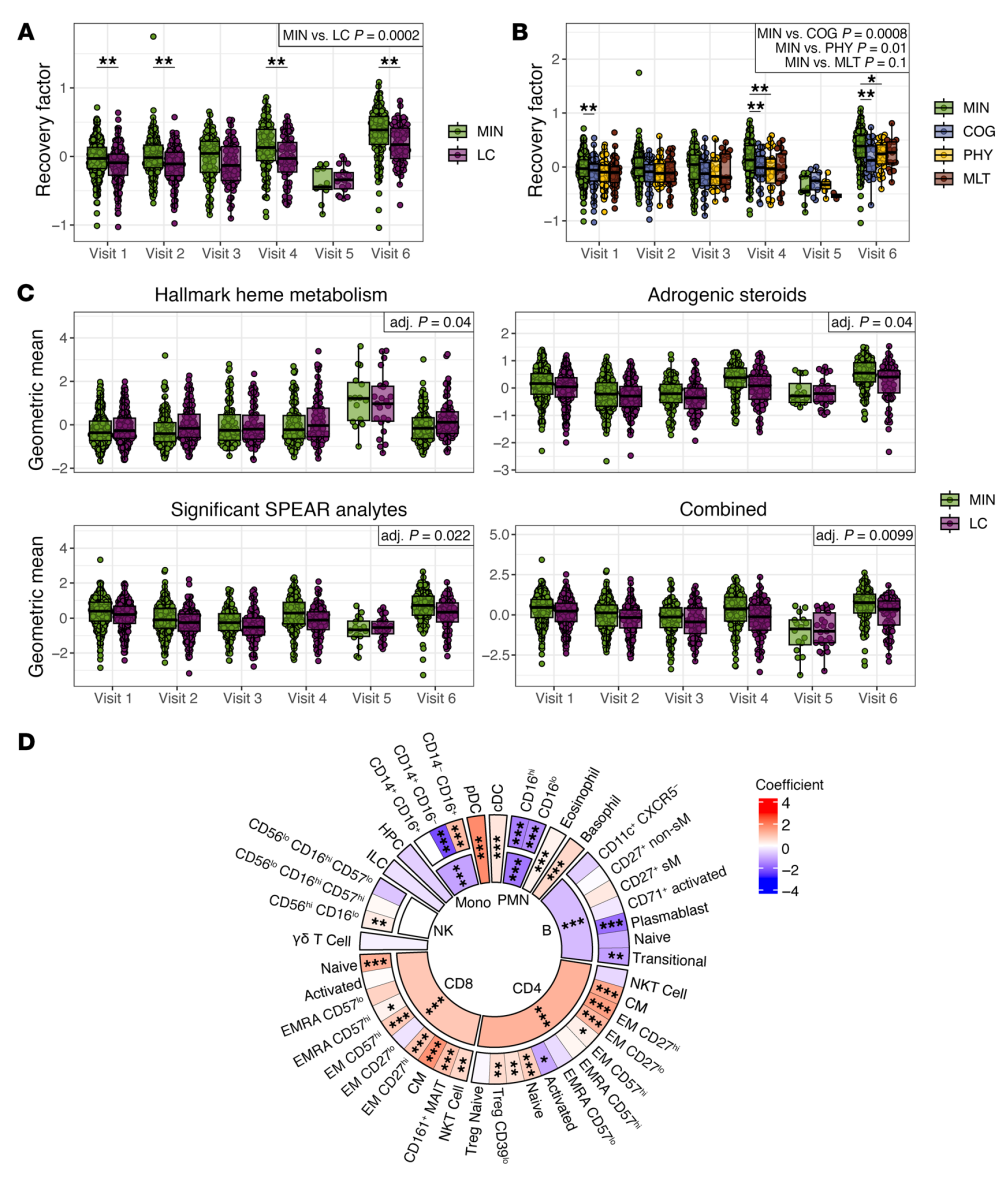
Recovery factor scores in acute phase data associate with eventual LC status. (**A**) Recovery factor scores during the acute disease phase for participants in the LC and MIN groups within 72 hours of hospital admission (visit 1) and at day 4 (visit 2), day 7 (visit 3), day 14 (visit 4), day 21 (visit 5), and day 28 (visit 6) after admission. (**B**) Recovery factor scores during the acute disease phase for participants in individual PRO clusters. (**C**) Geometric mean scores of analytes in enriched gene and metabolic sets and/or significant SPEAR analytes during the acute phase. No individual per-visit comparisons were significant after *P* value correction. *P* values in the top-right box in **A**–**C** show the significance of the recovery factor score or the geometric mean signature association with MIN versus LC or pairwise PRO cluster combinations. Bars above the box plots show the pairwise significance across groups in a per-visit comparison (*P < 0.05 and **P < 0.01). (**D**) Recovery factor score association with whole blood CyTOF immune cell populations during the acute phase (adj. *P < 0.05, adj. **P < 0.01, adj. ***P < 0.001). See also Supplemental Figure 9.

We next investigated whether the immune elements identified in the recovery factor were predictive of LC status when measured at the acute infection phase, prior to LC development. We computed recovery factor scores using immune profiling data from all participants in the convalescent cohort during their acute phase visits (visits 1–6, spanning the hospital admission through days 26–35 after admission). Remarkably, the recovery factor scores were significantly higher for MIN participants than for LC participants as early as at hospital admission (visit 1) and consistently higher during the acute period (Figure 5A and Supplemental Figure 9A). Recovery factor scores were also significantly higher for MIN groups than for COG groups and for MIN versus PHY groups in the acute phase when assessed across the 28-day time course (Figure 5B and Supplemental Figure 9B). Geometric mean scores of heme metabolism and androgenic steroid pathway analytes from the recovery factor, as well as the 26 significant SPEAR recovery factor analytes, were also significantly associated with LC status during the acute phase. The combined geometric mean score of analytes from these 3 feature groups in the acute phase data associated most significantly with MIN versus LC status (Figure 5C), as it did previously in the convalescent phase (Figure 3C).

We further assessed whether altered circulating immune cell composition in the acute phase could contribute to acute phase recovery factor scores. Association testing of recovery factor scores with whole blood CyTOF measurements during the acute phase showed that CD4^+^ and CD8^+^ T cells, conventional DCs (cDCs), plasmacytoid DCs (pDCs), eosinophils, basophils, and CD56^hi^ CD16^lo^ NK cells were significantly positively associated with the recovery factor scores (Figure 5D). Within– the CD4^+^ T cell compartment, naive, central memory T (Tcm), and effector memory T (Tem) subsets, as well as non-naive Tregs were significantly associated with recovery factor scores, while activated CD4^+^ T cells were inversely correlated. Within the CD8^+^ T cell compartment, naive, Tcm, and Tem subsets were positively associated with recovery factor scores, as were NKT cells and CD161^+^ MAIT cells. In contrast, monocytes, neutrophils, B cells, and plasmablasts in the acute phase were negatively associated with recovery factor scores (Figure 5D). These findings are consistent with a previous study that found higher plasmablast counts and lower total CD4^+^ T, total CD8^+^ T, CD4^+^ Tem, CD8^+^ Tem, Treg, NK, and DC counts in immune cell populations sampled on post-infection days 0–14 in patients with COVID-19 who experienced persisting symptoms at days 91–180 after infection (29). The similarities across both studies indicate an acute blood immune cell type signature of LC that is robust to variance in patient cohorts and LC definition.

In summary, our findings indicate that the major biologic signatures of the recovery factor that stratify LC from recovered participants in the convalescent phase — elevated heme metabolism gene signatures, reduced androgenic steroids, increased circulating inflammatory mediators, and increased monocytes and neutrophils — are evident early in the acute phase.

### Acute phase recovery factor scores distinguish acute disease severities and predict LC risk irrespective of acute severity.

**Figure 6:**
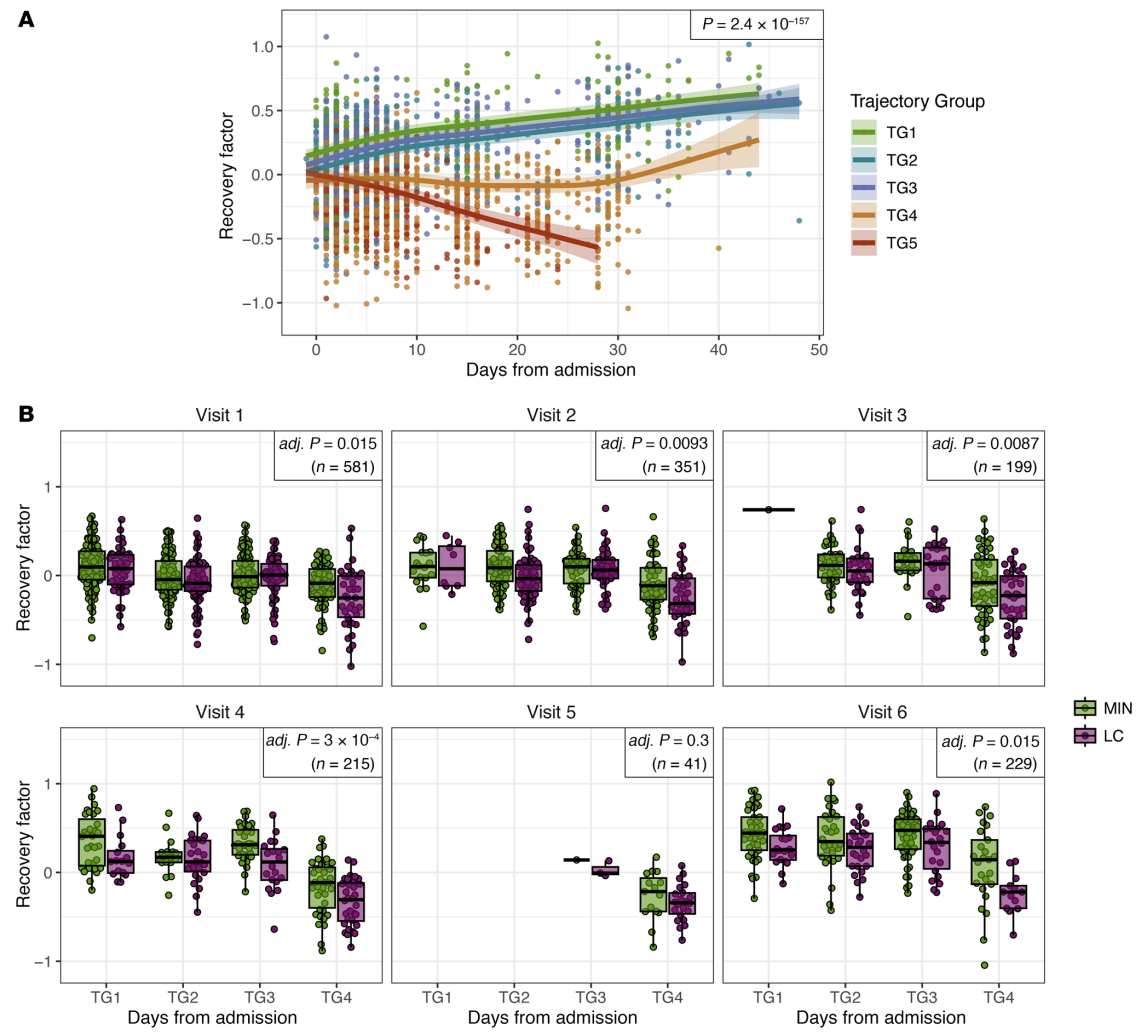
Recovery factor scores associate with acute disease phase trajectory groups, but identify LC irrespective of acute severity. (**A**) Longitudinal analysis of acute recovery factor scores for the full IMPACC cohort stratified by trajectory group (*n* = 1,148 participants). The *P* value shows the significance of the trajectory group term in a longitudinal model, correcting for age and sex as fixed effects and enrollment site and participant ID as random effects. (**B**) Recovery factor scores in the acute phase by convalescent MIN/LC label, stratified by acute trajectory group and visit number. *P* values show significance in distinguishing MIN versus LC labels in linear mixed models with sex, discretized admission age, and trajectory group as fixed effects and enrollment site and participant ID as random effects, performed separately for each acute visit and corrected across all visits.

We investigated the full IMPACC study cohort (*n* = 1,148 participants with at least 1 sample measurement at visit 1 for the omics modalities included in our model) to assess whether recovery factor scores determined from acute phase data would associate with patient severity trajectory group assignments. For this analysis, we included participants who did not survive beyond 28 days after hospital admission and participants without biospecimens and/or surveys during the convalescent phase. We found that the recovery factor scores were significantly associated longitudinally with acute disease trajectory groups and were highest in participants with milder disease courses (TG1–TG3), and were lowest in participants with the most severe acute disease trajectories (TG4 and TG5) (Figure 6A). Acute phase recovery factor scores increased over time for participants in all trajectory groups except TG5, the group with the most severe disease, in which participants died by day 28 after hospital admission (Figure 6A). To assess whether the association between acute recovery factor scores and convalescent LC status was simply due to acute recovery factor scores being an indicator of acute disease severity, we repeated the association test, including the trajectory group as a covariate at each visit (Figure 6B) and longitudinally (Supplemental Figure 9C). LC status was still significantly associated with acute recovery factor scores, even after taking the trajectory group into account. These findings suggest that recovery factor scores in the acute phase contain valuable information for predicting convalescent LC status beyond its correlation with acute disease severity.

### Machine learning models based on the recovery factor scores predict LC status during the convalescent phase.

We next assessed whether a combination of recovery factor scores and clinical characteristics (Supplemental Figure 10) could improve predictive performance. Machine learning models trained on acute phase recovery factor scores performed better than those trained on clinical features, and a model trained on the combination of both performed best (Supplemental Figure 11A). Similarly, machine learning models trained on both convalescent phase recovery factor scores and clinical features performed better than models trained on either alone (Supplemental Figure 11B). A model trained on the 26 SPEAR significant analytes was also predictive for LC, although at lower performance than models trained on the full recovery factor (Supplemental Figure 11B). This sparse model could be advantageous in a clinical setting as a diagnostic tool to identify individuals with LC. Notably, recovery factor scores at as early as visit 1 provided predictive performance (Supplemental Figure 11C), indicating that the recovery factor captures early predictive features of LC during the acute phase, albeit the signal at this early time point is not as strongly predictive as later in the convalescent phase.

## Discussion

In this study, we applied supervised multiomics integration methods to identify biologic features associated with LC in 513 participants from the IMPACC cohort. We took advantage of data availability from this cohort that was followed longitudinally after hospitalization for COVID-19 through 28 days of the acute disease phase and up to 1 year after discharge (18, 66). The IMPACC cohort is unique in its comprehensive inclusion of clinical data, biospecimens, and quarterly PRO surveys, combined with multiomics immunophenotyping at multiple time points throughout the acute and convalescent disease phases. A previous study of this cohort identified demographic and clinical risk factors associated with LC development, including female sex, comorbidities such as chronic heart, lung, and neurologic diseases, and a longer hospital stay (18). Here, we analyzed biologic data collected during the convalescent phase, which allowed us to identify a multiomics recovery factor capable of discriminating participants who recovered with minimal deficits from those who experienced clinical LC symptoms. Notably, we found that as early as 72 hours after hospital admission for COVID-19, recovery factor scores predict which patients, irrespective of their acute disease severity, will go on to experience LC. Biologic features associated with the recovery factor score indicate that reduced androgenic steroid levels, increased heme metabolism signatures, and persistent elevation of inflammation-associated serum proteins are hallmarks of LC that both identify individuals with LC during convalescence and predict which patients with acute COVID-19 will experience LC.

Increased levels of androgenic steroids in serum were positively associated with recovery factor scores and with participants who did not experience LC. Seven of the 12 leading-edge androgenic steroid metabolites were also included within the list of 26 analytes that were statistically significant within the recovery factor. Limited studies have elucidated the role of reduced androgenic steroids in LC, but, in agreement with our findings, lower testosterone levels have been associated with increased LC symptomatology in both men and women (31). Several intermediate metabolites in the canonical steroid hormone biosynthesis pathway were associated with the recovery factor and were decreased in participants with LC, including sulfated forms of testosterone precursors (pregnenolone and DHEA) and downstream metabolites (androsterone, epiandrosterone, and 5α-androstan-3β,17β-diol). DHEA-S, a leading-edge gene in the androgenic steroid pathway and a significant analyte in the recovery factor, exhibits immunosuppressive and antiinflammatory effects, particularly in neuroinflammation (70). In addition, testosterone can play an immunomodulatory role and is often reduced in patients with other critical illnesses (71). Thus, lower androgenic steroid levels may contribute to persistent inflammation in LC.

The list of top androgenic steroid metabolites in the recovery factor overlaps with the androgenic steroid signature from an all-female cohort of healthy controls compared with myalgic encephalomyelitis/chronic fatigue syndrome (ME/CFS) patients (72). All 6 androgenic steroid metabolites that were significantly elevated in healthy controls in the ME/CFS study [DHEA-S, androstenediol (3α,17α) monosulfate (2), androstenediol (3β,17β) disulfate (2), 5α-androstan-3β,17α-diol disulfate, androsterone sulfate, epiandrosterone sulfate] (72) were also part of the 26 significant SPEAR analytes, strongly implicating this signature in the shared symptomology, such as fatigue, postexertional malaise, and sleep disturbances, between ME/CFS and LC (73). In our study, a geometric mean score consisting of leading-edge metabolites from the androgenic steroids pathway significantly differentiated MIN versus LC in the entire cohort (Figure 3C and Figure 5C). Although ME/CFS criteria were not evaluated in our cohort, in part because similarities between LC and ME/CFS were not evident in 2020 when the cohort was enrolled, future studies could investigate whether features of the recovery factor are predictive for ME/CFS. The heme metabolism transcriptional signature in PBMCs was inversely associated with the recovery factor, such that it was elevated in participants with LC during the convalescent period. As with androgenic steroids, the leading-edge genes of the heme metabolism pathway were also expressed at higher levels during acute COVID-19 in participants who would later experience LC. Notably, overexpression of a heme metabolism signature in blood was recently reported in a separate cohort of 102 participants, including nonhospitalized and hospitalized patients with COVID-19 evaluated 1–3 months after infection (29), as well as in a cohort of nonhospitalized patients with COVID-19 (63), demonstrating that elevated heme metabolism is a common signature of LC across patients with diverse acute COVID-19 disease severities. In Hanson et al. (29), elevated heme metabolism was related to stress erythropoiesis induced by inflammation-associated anemia driven by IL-6–mediated hepcidin upregulation (29, 74). Participants who experienced LC had reduced iron and Hgb 2 weeks to 1 month after SARS-CoV-2 infection. This iron restriction was proposed not only to induce anemia, but also to impair lymphocyte function, which could delay the resolution of acute infection, resulting in sustained inflammation and thus persistent anemia that may partially explain the systemic symptomatology of acute COVID-19 and LC. Anemia of inflammation, also known as anemia of chronic disease, is a common complication associated with chronic inflammatory illnesses as well as ICU admission (75). In our study, anemia at hospital discharge was significantly negatively associated with the recovery factor, consistent with the prior finding that anemia as a complication was associated with LC (18). Moreover, Hgb and hematocrit measurements at hospitalization showed a significant positive association with the recovery factor (Figure 4A). Notably, expression levels of the leading-edge heme metabolism genes we identified in our study could also predict LC participants in the 2 independent cohorts discussed above (29, 63), validating the relevance of the heme metabolism signature identified in the recovery factor, and demonstrating its ability to predict which patients will experience LC as early as the acute phase of COVID-19. Anemia status prior to infection was not available for the IMPACC cohort, nor was it assessed in the 2 cohorts in which we validated our heme metabolism signature (29, 63). Interestingly, an assessment of LC and preexisting comorbidities, identified by self-reporting questionnaires, found that preexisting anemia was associated with a decreased risk of developing LC (76). This finding is consistent with a model in which SARS-CoV-2 infection drives the inflammation that induces the anemia associated with LC. However, future studies are needed to evaluate whether preexisting anemia is a risk or protective factor for LC.

We also found evidence of persistent inflammation in participants with LC. Inflammation-associated serum factors, such as CXCL9, CSF1, and FGF21, were identified by SPEAR as significant analytes in the recovery factor, however, they did not reach significance when associated individually with LC status. Of note, FGF21 levels measured in the acute phase were previously associated with cognitive and multidomain deficit PRO clusters relative to MIN in this cohort (18), and FGF21 has been proposed as a biomarker for chronic inflammation in ME/CFS (77), a complex chronic disease that overlaps clinically with LC. Furthermore, LRG1, which is activated by the inflammatory IL-6/STAT3 pathway, is significantly elevated in participants with LC and possibly contributes to vascular pathology in LC (55–57). Although IL-6 did not reach significance in the SPEAR factor (SPEAR Bayesian posterior selection probability = 0.86), it was within the top 50 analytes, perhaps promoting anemia and increased heme metabolism signatures, as discussed above. Also, recovered participants demonstrated elevated levels of the tissue repair protein DNER, consistent with other cohorts (28). Together, these findings point to inefficient tissue repair, as well as persistent inflammation, inducing anemia and stress erythropoiesis, as key drivers of LC.

Across all time points, several immune cell subsets were consistently associated with the recovery factor. In both the acute and convalescent phases, CD161^+^ MAIT cell frequencies were positively associated with the recovery factor, while monocyte, neutrophil, and CD14^+^CD16^–^ classical monocyte frequencies were negatively associated with the recovery factor. CD14^+^ monocytes can induce CD8^+^ T cells to produce high levels of IFN-γ in patients with LC (78). The consistent association of classical monocytes with low recovery factor scores suggests that they contributed to inflammation starting in the acute disease phase and continuing into convalescence in participants who experienced LC. In contrast, the B cell population was negatively associated with the recovery factor during acute disease, but positively associated during convalescence. A prior study with limited participants reported successful treatment of LC with intravenous IgG (IVIG) administration (79), suggesting that restoring homeostasis of B cells and subsequent IgG production should be evaluated for treating LC. An ongoing clinical trial assessing IVIG for the treatment of neurological LC (ClinicalTrials.gov, NCT05350774) will provide further insights. Our data on immune cell frequencies in the recovery factor agree with several previous reports of individuals with LC. The positive association of CD4^+^ and CD8^+^ T cells, pDCs, and cDCs with the recovery factor during acute disease, with a negative association of monocytes, neutrophils, and B cells, indicates reduced T cell immunity relative to inflammatory innate immunity in individuals susceptible to LC. This finding is in keeping with the cellular trends observed by Hanson et al. in acute disease (29), and in men during convalescence by Silva and colleagues (31). Consistent with the findings of Klein et al. (26), we did not find significant associations of the recovery score with naive CD4^+^ or naive CD8^+^ T cells in the convalescent phase of COVID-19. Although whole blood single-cell RNA-Seq data were not available for this cohort, transcriptomics profiles of individual cell subsets, such as monocytes and neutrophils, could provide further insight into the pathways by which they contribute to LC.

A previous study from IMPACC identified a “severity factor” that significantly associated with clinical outcomes during acute COVID-19 (39). Given that both the severity factor and the recovery factor are associated with inflammatory signatures during acute COVID-19, we compared immune cell types associated with the recovery factor with those associated with the severity factor at this stage of disease. There were notable similarities: for example, monocytes, B cells, and neutrophils are negatively associated with the recovery factor and positively associated with the severity factor. Likewise, CD4^+^ and CD8^+^ T cells are positively associated with the recovery factor and negatively associated with the severity factor. These cellular associations suggest that inefficient adaptive immunity during acute disease, with elevated frequencies of inflammatory innate cells, contributes to LC susceptibility. This model is consistent with our past (18) and current findings in the IMPACC cohort that reduced anti–SARS-CoV-2 antibody levels and increased viral titers in patients within the first 72 hours of hospital admission were associated with participants who would go on to experience LC (Supplemental Figure 10, B and C). These findings are also consistent with other reports showing that hospitalized patients are more susceptible to LC than are nonhospitalized individuals (14, 80). Nonetheless, our recovery factor predicts which patients will experience LC irrespective of acute disease severity, indicating that the model has learned features of COVID-19 beyond inflammation that are associated with COVID-19 severity.

Although symptom groups, such as respiratory symptoms, were found to be significantly associated with LC in this cohort (18), the multiomics recovery factor does not associate with a particular clinical symptom group. Instead, it captures biomarkers predictive of global physical deficits, as reported by patients after acute COVID-19. While assessing the entire multiomics SPEAR factor in convalescent patients is impractical, our findings indicate that assessing the 26 significant SPEAR analytes would aid in LC diagnoses (Supplemental Figure 11B). Future studies with even larger datasets would be needed to identify the LC mechanisms specific for distinct endotypes of disease.

This study has several limitations. The reliance on self-reported survey data to identify symptoms and classify participants into MIN/LC and individual PRO clusters may have introduced potential biases. To address this, population-normalized PRO survey scores were utilized (81–83), and comparisons were made with pre-illness health status when possible. Additionally, our study was designed early in the pandemic (March 2020), before the full spectrum of LC symptoms was characterized, and thus the surveys did not capture current commonly recognized manifestations such as brain fog, fatigue, sleep disturbances, neuropathy, and dysautonomia. Patients were also not evaluated clinically for pain, sensory sensitivity, or other clinical features of chronic disease during convalescence, when surveys were instead administered, so we cannot determine if the recovery factor correlates with clinical features of chronic disease. Self-selection bias may also be present, as patients with severe LC symptoms might have been less likely to respond to the surveys. As the study cohort was recruited during the early phases of the pandemic (May 2020 through March 2021), it consists of individuals infected only with the original SARS-CoV-2 strain and does not include data on subsequent variants of concern; however, we note that the recovery factor heme metabolism signature identified LC patients in a cohort infected with the Omicron variant (63) (Supplemental Figure 6D). Vaccination data were self-reported and limited to the post–acute phase, since enrollment was largely completed prior to vaccine rollout, and exact vaccination dates were unavailable for some participants. Furthermore, as part of the study design, all participants in the IMPACC cohort were hospitalized for COVID-19. Consequently, the multiomics factors were constructed without incorporating profiles from patients with COVID-19 with mild disease, potentially introducing a bias toward those with severe disease. Nonetheless, we note that participants who recovered after hospitalization without experiencing LC (MIN group) had a median Physical PRO score that was slightly better (>50) than the overall population norm (Supplemental Figure 3A), indicating that the severe nature of disease experienced by this hospitalized cohort did not bias health in the convalescent period to worse than population norms. We are not aware of a dataset from a nonhospitalized or asymptomatic cohort with the same depth of OMICs measurements needed to reconstruct the entire recovery factor to test if predictive power is maintained in nonhospitalized cohorts. However, we validated that elevated heme metabolism scores, a key biologic pathway in the recovery factor, were associated with LC in a cohort that included hospitalized and nonhospitalized patients with COVID-19 (29), as well as in a cohort consisting of nonhospitalized patients with COVID-19 (63). While it remains possible that biologic signatures of LC exclusive to COVID-19 patients with mild acute disease may not be included in the recovery factor, validation of the heme metabolism signature in these independent cohorts suggests that the molecular signatures of LC identified in our hospitalized cohort likely extend to nonhospitalized and asymptomatic SARS-CoV-2–infected individuals.

Despite these limitations, the study possesses multiple strengths. In addition to the prospective design, with acute and convalescent longitudinal multiomics profiling, enrollment through multiple sites across the United States enhances the broad representation of the cohort and mitigates potential participant recruitment biases, contributing to the robustness of the findings.

In conclusion, supervised multiomics factor construction of immune profiling data from SARS-CoV-2–infected participants who recovered with minimal deficits or experienced LC indicates that peripheral blood leukocytes and serum factors associated with inflammation, reduced androgenic steroids, and elevated heme metabolism signatures predict which participants will experience LC, irrespective of acute disease severity. Moreover, these signatures are maintained into convalescence, indicating that persistent inflammation driving anemia is likely a key contributor to LC. Further studies will be needed to determine why inflammation persists in some patients with COVID-19. We did not assess persistent SARS-CoV-2 viral loads or viral reactivation; however, a recent IMPACC study (84) suggests that latent virus reactivation may contribute to LC, consistent with other studies (26, 30, 31). Altogether, our data, paired with prior congruent reports, suggest that impaired lymphocyte function early in COVID-19 reduces cellular and humoral adaptive immunity and contributes to high SARS-CoV-2 viral loads. Elevated viral loads can trigger innate immune cell responses that increase inflammatory cytokines, driving inflammation-associated anemia that further reduces lymphocyte function, which could enable reactivation of latent viruses. Such unresolved persistent inflammation likely leads to LC pathology. Strategies to break the cycle of inflammation and correct the inflammation-associated anemia may promote recovery from LC and merit further investigation.

## Methods

### Sex as a biological variable.

Our study examined male and female participants. The cohort included 310 (60%) men and 213 (40%) women. Female sex was negatively associated with the recovery factor, in line with a higher proportion of women experiencing LC. To control for this imbalance, sex and age were used as covariates in statistical testing to identify robust trends for both sexes unless otherwise stated.

### Study design.

The IMPACC Cohort consists of 1,164 patients hospitalized with COVID-19 from 20 US hospitals (15 academic institutions), enrolled within 72 hours of admission between May 2020 and March 2021. Participants with confirmed positive SARS-CoV-2 PCRs were followed during the acute infection phase (1–28 days after admission) and the convalescent phase (3–12 months after discharge). Clinical data (e.g., hospital stay, comorbidities, complications, mortality) and biological samples were collected during the acute phase, whereas standardized PRO surveys were assessed quarterly over the convalescent phase (18, 37, 38). Six validated surveys, including the PROMIS physical function, cognitive function, global mental health, psychosocial illness impact, and dyspnea time extension (80, 81) and the EQ-5D-5L (82) were used to evaluate general health and deficits in specific domains. Also, overall health recovery scores compared post-discharge function with pre-infection status (18). Full study design details are available in the Supplemental Methods.

### Statistics.

Unless otherwise stated, multiple comparisons were accounted for via Benjamini-Hochberg correction, with adjusted *P* values of less than 0.05 considered significant. Linear mixed-effects modeling was used for differential analysis of multiomics factors, analyte geometric means, and individual analytes across LC groups, after adjusting for sex, age, and visit number as fixed effects and enrollment site as a random effect. Generalized mixed-effects modeling was used to investigate longitudinal variations across conditions using samples from all visits after further adjusting for participant ID as a random effect. These sets of fixed and random effects for baseline and longitudinal samples were the default for all mixed-effects modeling, unless otherwise stated. See Supplemental Methods for detailed descriptions of all statistical analyses and models.

### Study approval.

NIAID staff conferred with the Department of Health and Human Services Office for Human Research Protections (OHRP) regarding the potential applicability of the public health surveillance exception (45CFR46.102) to the IMPACC study protocol. OHRP concurred that the study satisfied criteria for the public health surveillance exception, and the IMPACC study team sent the study protocol and participant information sheet for review and assessment to IRBs at participating institutions. Twelve institutions elected to conduct the study as public health surveillance, while 3 sites with prior IRB-approved biobanking protocols elected to integrate and conduct IMPACC under their institutional protocols (University of Texas at Austin, IRB 2020-04-0117; UCSF, IRB 20-30497; Case Western Reserve University, IRB STUDY20200573) with informed consent requirements. Participants enrolled under the public health surveillance exclusion were provided information sheets describing the study, samples to be collected, and plans for data deidentification and use. Those who requested not to participate after reviewing the information sheet were not enrolled. In addition, individuals did not receive compensation for study participation while inpatient and were subsequently offered compensation during outpatient follow-ups.

### Data availability.

Data used in this study are available in the ImmPort repository under accession number SDY1760 and in the Database of Genotypes and Phenotypes (dbGaP) under accession number phs002686. All code used in this study has been deposited in Bitbucket (https://bitbucket.org/kleinstein/impacc-public-code/src/master/multiomics-longcovid/). The raw data for all figures are available in the Supporting Data Values file.

## Author contributions

PMB, SHK, LG, LIRE conceived the study. AH, CS, B Peters, and JDA curated the data. GG, JM, JPG, JFM, AH, CS, TC, and NDJ conducted formal analysis. The IMPACC Network acquired fund¬ing and provided resources. AO, ACS, ADA, AFS, CB, CBC, CLH, CSC, DAH, DBC, DJE, EFR, EKH, EM, F Kheradmand, F Krammer, GAM, HS, HTM, JPM, JS, KCN, KW, LRB, MAA, MK, MMD, NIAH, NR, RPS, SCB, SEB, SKS, VS, WBM, and WE provided data and resources. GG, JM, JPG, JFM, SHK, and LG designed the study methodology and code. SF, MCA, OL, KKS, RRM, JDA, SHK, LG, and LIRE supervised the study. All authors wrote, edited, and reviewed the manuscript. Co–first authorship of GG, JM, JPG, and JFM was assigned on the basis of their contributions to the work.

## Funding support

This work is the result of NIH funding, in whole or in part, and is subject to the NIH Public Access Policy. Through acceptance of this federal funding, the NIH has been given a right to make the work publicly available in PubMed Central.

- NIAID, NIH (3U01AI167892-03S2, 3U01AI167892-01S2, 5R01AI135803-03, 5U19AI118608-04, 5U19AI128910-04, 4U19AI090023-11, 4U19AI118610-06, R01AI145835-01A1S1, 5U19AI062629-17, 5U19AI057229-17, 5U19AI057229-18, 5U19AI125357-05, 5U19AI128913-03, 3U19AI077439-13, 5U54AI142766-03, 5R01AI104870-07S1, 3U19AI089992-09, 3U19AI128913-03, and 5T32DA018926-1, 3U19AI1289130, U19AI128913-04S1, R01AI122220).
- NIH (UM1TR004528).
- NSF (DMS2310836).

## Supplementary Material

Supplemental data

ICMJE disclosure forms

Supporting data values

## Version 1 09/09/2025

In-Press Preview

## Version 2 11/03/2025

Electronic publication
