# Supplementary material for: A multiomics recovery factor predicts long COVID in the IMPACC study
Source: J Clin Invest. 2025 Sep 9;135(21):e193698. doi: 10.1172/JCI193698 (PMC12582403; doi:10.1172/JCI193698)
Supplement: Supplemental data [file jci-135-193698-s007.pdf]

SUPPLEMENTAL MATERIAL

TABLE OF CONTENTS

SUPPLEMENTAL MATERIAL 1
EXPERIMENTAL MODEL AND STUDY PARTICIPANT DETAILS 3
Study design 3
PRO group assignments 3
IMPACC Convalescent cohort definition 3
METHOD DETAILS 5
Sample processing and quantification and batch randomization 5
Train and test cohort split 5
Data preprocessing and additional quality control 5
PBMC Gene Expression (PGX) 6
Serum Olink (SO) 7
Plasma Proteomics Global and Targeted (PPG, PPT) 7
Plasma Metabolomics Global (PMG) 7
Whole Blood CyTOF cell frequencies (CyTOF) 7
Whole Blood CyTOF marker MSI (BCT) 8
Clinical data processing 8
Acute infection trajectory group definition 8
Vaccination data collection 8
Antibody titers 9
Viral load 9
MOFA model construction 9
SPEAR model training 9
Data imputation 9
SPEAR model training for each response variable 9
SPEAR model selection and evaluation 10
Computing factor scores for the Test cohort and acute phase visits immune profiling 10
Statistical analysis and association analysis 10
Factor scores and geometric mean scores association 10
Factor scores and geometric mean scores longitudinal association 11

Blood CyTOF cell frequencies longitudinal association 11
Effect of vaccination on factor scores 11
Factor annotation and enrichment analysis 11
Geometric mean score 12
Heme Metabolism validation in the Hanson et al. (2024) and Karisola et al. (2025) cohorts 12
Machine learning approach to compare recovery factor and clinical models 13
SPEAR acute and convalescent models 13
SPEAR significant analytes model... 13
Clinical model construction 13
Model training, evaluation, and performance comparison 13
SUPPLEMENTAL FIGURES 14
CONFLICT OF INTEREST 26
SUPLEMENTAL ACKNOWLEGMENTS 28
SUPPLEMENTAL REFERENCES 32

EXPERIMENTAL MODEL AND STUDY PARTICIPANT DETAILS

Study design

The IMPACC Cohort consists of 1164 participants admitted to 20 US hospitals (affiliated with 15 academic institutions) between May 2020 and March 2021 that were enrolled within 72h of hospital admission for COVID-19 infection. Participants with confirmed positive SARS-CoV-2 PCR and COVID-19 infection symptomatology were followed longitudinally during the acute infection phase (1-28 days after hospital admission) and the convalescent phase (3 months to 12 months after hospital discharge). Details on the study design for clinical data and biological sample collection were previously described (1–3). Clinical data including length of hospital stay, complications, mortality, and other pre-defined outcomes were collected over the acute phase. Self-reported symptoms, reinfections, SARS-CoV-2 vaccination, re-hospitalizations, and standardized patient-reported outcome surveys were assessed quarterly over the convalescent period through a mobile application and remote visits (1). The surveys at these remote visits captured upper respiratory symptoms, cardiopulmonary symptoms, systemic symptoms, neurologic symptoms, and gastrointestinal symptoms. Additionally, six validated Patient-Reported Outcome (PRO) surveys were used to evaluate general health and deficits in specific health domains including EQ-5D-5L (4), and the Patient-Reported Outcomes Measurement Information System (PROMIS) (5,6) Physical function, Cognitive function, Global Health Mental, Psychosocial Illness Impact and Dyspnea Time Extension surveys. Overall health was also assessed by a health recovery score to indicate overall pot discharge mental and physical function compared to pre-COVID function (18).

PRO group assignments

Participants were assigned to PRO groups as previously described (1). Briefly, all six PRO scores were standardized using standard PROMIS T-scores or a standard value set. The health recovery score was computed utilizing a Visual Analog Scale ranging from 1 to 100. The individual PRO scores collected over the convalescent period were modelled longitudinally using Latent Class Mixed Models (LCMM). A Ward clustering analysis was then applied to group participants with similar PRO longitudinal patterns. Four distinct clusters were identified, and t-statistics were calculated comparing the mean value of individual PRO scores within each cluster versus the mean value of that PRO across the remaining clusters. After association with the specific PROs, clusters were labeled minimal deficit (MIN), physical predominant (PHY), mental/cognitive predominant (COG), and multi/pan domain deficit (MLT).

IMPACC Convalescent cohort definition

In this study, we only included participants who survived hospitalization and had at least one quarterly set of surveys post discharge and immune profiling measurements for at least one of the following assays during the convalescent phase (visits 7-10 at 3, 6, 9, and 12 months post hospital discharge): serum O-link, plasma targeted proteomics, plasma global proteomics, plasma global metabolomics, PBMC transcriptomics and CyTOF measurements, yielding an analysis cohort of 513 participants (Table S1).

Table S1. Demographics and baseline characteristics of the Convalescent cohort

		**Overall** (N=513)	**MIN** (n=318)	**LC** (n=195)	**Overall p-value**
Age at enrollment (years), median (IQR)	(n=513)	57.0 (19.0)	56.5 (20.0)	57.0 (16.0)	0.653
Sex at birth	Male	310 (60%)	213 (67%)	97 (50%)	<.001
	Female	203 (40%)	105 (33%)	98 (50%)	
Race	White	248 (48%)	157 (49%)	91 (47%)	0.352
	Black	118 (23%)	73 (23%)	45 (23%)	
	Other	101 (20%)	56 (18%)	45 (23%)	
	Asian	19 (4%)	13 (4%)	6 (3%)	
	Multiple	7 (1%)	6 (2%)	1 (1%)	
	American Indian/Alaska Native	6 (1%)	2 (1%)	4 (2%)	
	Native Hawaiian/Pacific Islander	4 (1%)	3 (1%)	1 (1%)	
	Unknown	10 (2%)	8 (3%)	2 (1%)	
Hispanic ethnicity	Non-Hispanic	334 (65%)	208 (65%)	126 (65%)	0.717
	Hispanic	168 (33%)	102 (32%)	66 (34%)	
	Unknown	11 (2%)	8 (3%)	3 (2%)	
Comorbidities	Hypertension	282 (55%)	163 (51%)	119 (61%)	0.031
	Diabetes	168 (33%)	94 (30%)	74 (38%)	0.049
	Chronic respiratory (not asthma)	85 (17%)	37 (12%)	48 (25%)	<.001
	Asthma	90 (18%)	51 (16%)	39 (20%)	0.252
	Chronic cardiac disease	122 (24%)	62 (19%)	60 (31%)	0.004
	Chronic kidney disease	62 (12%)	42 (13%)	20 (10%)	0.32
	Malignant neoplasm	37 (7%)	26 (8%)	11 (6%)	0.281
	Chronic neurologic disorder	49 (10%)	20 (6%)	29 (15%)	0.001
	Liver disease	25 (5%)	12 (4%)	13 (7%)	0.14
	History of SOT or BMT	38 (7%)	18 (6%)	20 (10%)	0.054
	Current or former smoking and/or vaping	151 (29%)	86 (27%)	65 (33%)	0.129
	Substance use (drugs, alcohol, and/or cannabis)	35 (7%)	20 (6%)	15 (8%)	0.541
BMI Category	Underweight	4 (1%)	4 (1%)	0 (0%)	0.003
	Normal weight	54 (11%)	34 (11%)	20 (10%)	
	Overweight (25.1-29.9)	141 (27%)	95 (30%)	46 (24%)	
	Class 1-2 Obesity (30-39.9)	220 (43%)	135 (42%)	85 (44%)	
	Class 3 Obesity (40+)	80 (16%)	37 (12%)	43 (22%)	
	Missing	14 (3%)	13 (4%)	1 (1%)	
Number of comorbidities	0	34 (7%)	29 (9%)	5 (3%)	<.001
	1	82 (16%)	62 (19%)	20 (10%)	
	2	100 (19%)	66 (21%)	34 (17%)	
	3	101 (20%)	55 (17%)	46 (24%)	
	4	69 (13%)	39 (12%)	30 (15%)	
	5	127 (25%)	67 (21%)	60 (31%)	
Infiltrates on chest X-ray or chest tomography	No infiltrates	110 (22%)	67 (22%)	43 (23%)	0.978
	Unilateral infiltrates	45 (9%)	29 (10%)	16 (9%)	
	Bilateral infiltrates	331 (68%)	206 (68%)	125 (68%)	
	Unknown/Missing	3 (1%)	2 (1%)	1 (1%)	
Level of respiratory support	Mechanically ventilated, or ECMO (OS=6)	33 (6%)	20 (6%)	13 (7%)	0.022
	Non-invasive ventilation, or high flow nasal O2 (OS=5)	77 (15%)	58 (18%)	19 (10%)	
	Supplemental oxygen (not high flow) (OS=4)	290 (57%)	180 (57%)	110 (56%)	
	None (OS=3)	113 (22%)	60 (19%)	53 (27%)	
SpO2/FiO2 ratio category at lowest sat	235 or lower	84 (16%)	62 (19%)	22 (11%)	0.077
	236-315	96 (19%)	57 (18%)	39 (20%)	
	315 or higher	304 (59%)	184 (58%)	120 (62%)	
	Missing	29 (6%)	15 (5%)	14 (7%)	
SOFA Score, median (IQR)	(n=513)	0.0 (2.0)	0.0 (2.0)	0.0 (2.0)	0.991
Lymphocyte count (1000s/microliter), median	(n=431)	1.0 (0.9)	1.0 (0.8)	1.2 (1.1)	0.234
Platelets (1000s/microliter), median (IQR)	(n=487)	239.0 (124.0)	239.0 (126.0)	238.0 (125.5)	0.023
ALT (Units/L), median (IQR)	(n=453)	32.0 (34.0)	33.5 (34.0)	32.0 (32.0)	0.271
Creatinine (mg/dL), median (IQR)	(n=494)	0.9 (0.4)	0.9 (0.4)	0.8 (0.4)	0.596
CRP (mg/L), median (IQR)	(n=375)	13.0 (61.1)	13.2 (55.0)	12.3 (64.1)	0.23
D-dimer (mg/L), median (IQR)	(n=369)	0.7 (0.8)	0.8 (0.9)	0.6 (0.6)	0.063
Troponin (ng/mL), median (IQR)	(n=170)	0.0 (0.1)	0.0 (0.1)	0.0 (0.1)	0.899
Length of stay (days), median (IQR)	(n=500)	5.0 (5.0)	5.0 (6.0)	6.0 (5.0)	0.206
ICU at any time during acute hospitalization	Yes	134 (26%)	84 (26%)	50 (26%)	0.846
Acute trajectory group	1	126 (25%)	83 (26%)	43 (22%)	0.254
	2	167 (33%)	95 (30%)	72 (37%)	
	3	141 (27%)	86 (27%)	55 (28%)	
	4	79 (15%)	54 (17%)	25 (13%)	
Complications	Any complications	446 (87%)	284 (89%)	162 (83%)	0.042
	Number of complications, median (IQR) n=513	2.0 (2.0)	2.0 (2.0)	2.0 (2.0)	0.367
	Acute renal injury/ failure	84 (16%)	50 (16%)	34 (17%)	0.611
	Liver dysfunction/ failure	63 (12%)	45 (14%)	18 (9%)	0.099
	Anemia	57 (11%)	31 (10%)	26 (13%)	0.21
	Shock (use of vasopressors)	37 (7%)	18 (6%)	19 (10%)	0.083
	Bacteremia	41 (8%)	27 (8%)	14 (7%)	0.595
	Atrial fibrillation	25 (5%)	15 (5%)	10 (5%)	0.834
	Acute venous thromboembolism	25 (5%)	14 (4%)	11 (6%)	0.527
	Congestive heart failure (CHF) / cardiomyopathy	22 (4%)	12 (4%)	10 (5%)	0.462
	Hyperglycemia	20 (4%)	11 (3%)	9 (5%)	0.511
Medications	Remdesivir	332 (65%)	211 (66%)	121 (62%)	0.322
	Steroids	354 (69%)	229 (72%)	125 (64%)	0.06
Convalescent symptoms	Any symptom reported during convalescence	273 (53%)	140 (44%)	133 (68%)	<.001
	Any upper respiratory	91 (18%)	34 (11%)	57 (29%)	<.001
	Conjunctivitis/red eyes	65 (13%)	23 (7%)	42 (22%)	<.001
	Sore throat	39 (8%)	13 (4%)	26 (13%)	<.001
	Any cardiopulmonary	186 (36%)	87 (27%)	99 (51%)	<.001
	Dyspnea	155 (30%)	69 (22%)	86 (44%)	<.001
	Cough	106 (21%)	43 (14%)	63 (32%)	<.001
	Any systemic	154 (30%)	61 (19%)	93 (48%)	<.001
	Fatigue	96 (19%)	31 (10%)	65 (33%)	<.001
	Myalgia	106 (21%)	36 (11%)	70 (36%)	<.001
	Fever	14 (3%)	6 (2%)	8 (4%)	0.135
	Chills	24 (5%)	9 (3%)	15 (8%)	0.011
	Any neurologic	143 (28%)	59 (19%)	84 (43%)	<.001
	Headache	98 (19%)	35 (11%)	63 (32%)	<.001
	Anosmia	76 (15%)	29 (9%)	47 (24%)	<.001
	Any gastrointestinal	36 (7%)	11 (3%)	25 (13%)	<.001

METHOD DETAILS

Sample processing and quantification and batch randomization

Biological sample collection and processing was homogeneously performed by every participating academic center. Batch randomization, sample processing and assay preparation and quantification were performed according to previously published protocols (2,7,8).

Train and test cohort split

Participants in the convalescent cohort were split into 80% Train cohort and 20% Test cohort, maintaining the proportions of PRO group individuals with each group.

Data preprocessing and additional quality control

Data preprocessing included sample filtering according to quality control steps, feature filtering to remove features with a high percentage of missing values or low variance, batch correction, missing value imputation and data transformation (Table S2). We evaluated the influence of potential batch effects on the different assays using Principal variance component analysis (PVCA). The pre-processing steps for each data modality are described in detail below. Train and Test cohort datasets were independently processed to avoid data leakage between the datasets. For the feature filtering step, the same features were filtered on the Test cohort as identified on the Train cohort, to allow for the Factor score reconstruction.

Table S2. Summary of data preparation steps. For each assay, we first filtered out samples according to the sample filtering criteria and features based on the feature filtering criteria. N/A: no additional step taken. Pareto-scaling: in-house function of normalizing each centered variable by the square root of the standard deviation. Half-min: missing values were replaced using half the minimum of observed values for the corresponding feature.

Assay name	Sample filtering	Feature filtering	Additional batch correction	Missing value imputation	Data transformation
PBMC gene expression (PGX)	Passed & questionable QC	Protein coding, Genes with CPM >=1 in >5% of samples in a PRO group, top 20% highly variable genes	RemoveBatchEffects (limma)	N/A	Log2CPM and scaling
Serum O-link (SO)	Passed & questionable QC	Remove samples with all missing values and features with >99%	N/A	N/A	Scaling

		missing values			
Plasma proteomics global and targeted (PPG & PPT)	Passed & questionable QC	Removed features with >99% missing values	Batch correction (ComBAT)	Half-min	Median normalization in linear space Log2 transformation with pseudocount of 1 and scaling
Plasma metabolomics global (PMG)	Passed & questionable QC	Removed non-xenobiotic features with IQR=0		Half-min	Pareto-scaling
Blood CyTOF	Passed & questionable QC	Removed undefined features, debris, multiplets, platelets, RBC. Removed features with all missing values, and samples with >80% missing values.	N/A	N/A	Log1p transformation of blood cytof frequencies followed by scaling of cell subset across samples.
Blood CyTOF MSI (BCT)	Passed & questionable QC	Removed undefined features, debris, multiplets, platelets, RBC. Removed features with all missing values, and samples with more than 80% missing values.	RemoveBatchEffects (limma)	N/A	Scaling

PBMC Gene Expression (PGX)

We filtered for protein-coding genes and removed genes with low expression (genes that did not exceed the threshold of counts per million (CPM) >= 1 in more than 5% of samples in at least one

outcome group (PRO group). We also selected the top 20% highly variable features by mean absolute deviation (MAD) of the log-transformed CPM values. The count data was transformed using the *voom* function from the limma R package (9), batch correction was performed to remove the effects of technical variables, including sample processing batch (phase) and library preparation plate using the function *removeBatchEffect* from limma, and the data was scaled.

Serum Olink (SO)

Samples with all missing values and features with >99% missing values were removed, and the data was scaled.

Plasma Proteomics Global and Targeted (PPG, PPT)

Features with more than 99% missing values were removed, and the data was median normalized in lineal space, imputed using the half-min approach (missing values were replaced using half the minimum of observed values for the corresponding feature), log-transformed using a pseudocount of 1. Batch correction to correct for the effects of processing batches (phase) and plates was performed with the *ComBat* function (10) of the sva R package. Finally, data was scaled.

Plasma Metabolomics Global (PMG)

Missing values were imputed using the half-min approach, non-xenobiotic features with IQR=0 were removed as previously described (7), and the data was pareto-scaled.

Whole Blood CyTOF cell frequencies (CyTOF)

Cells were manually labeled into parent and child subsets as previously described (7) (Table S3). Cells annotated as cellular debris, multiplets, platelets, red blood cells, and undefined populations were removed. Cellular population counts were converted to a normalized frequency by dividing by the total counts per sample. For non-granulocyte cell types, the total counts normalization was calculated without granulocytes. The normalized frequencies were then log1p transformed and scaled by cell subset across samples.

Table S3. Parent and child cell type annotations used in the whole blood CyTOF data.

Parent Cell Type	Child Cell Types
B Cell	B Cell (CD27+ non-switched memory), B Cell (CD27+ switched memory), B Cell (CD71+ activated), B Cell (Plasmablast), B Cell (naive), B Cell (transitional)
CD4+ T Cell	CD4+ NKT Cell, CD4+ T Cell (CM), CD4+ T Cell (EM CD27hi), CD4+ T Cell (EM CD27low), CD4+ T Cell (EM CD57hi), CD4+ T Cell (EMRA CD57hi), CD4+ T Cell (EMRA CD57low), CD4+ T Cell (activated), CD4+ T Cell (naive), CD4+ Treg (CD39hi), CD4+ Treg (CD39low), CD4+ Treg (naive)
CD8+ T Cell	CD8+ NKT Cell, CD8+ T Cell (CD161+ MAIT), CD8+ T Cell (CM), CD8+ T Cell (EM CD27hi), CD8+ T Cell (EM CD27low), CD8+ T Cell (EM CD57hi), CD8+ T Cell (EMRA CD57hi), CD8+ T Cell (EMRA CD57low), CD8+ T Cell (activated), CD8+ T Cell (naive)
γδ T Cell	γδ T Cell
NK Cell	NK Cell (CD56hi CD16low), NK Cell (CD56low CD16hi CD57hi), NK Cell (CD56low CD16hi CD57low)
Innate Lymphoid Cell	Innate Lymphoid Cell

Hematopoietic Progenitor Cell	Hematopoietic Progenitor Cell
Monocytes	Monocytes (CD14+CD16+), Monocytes (CD14+CD16-), Monocytes (CD14-CD16+)
Neutrophil	Neutrophil (CD16hi), Neutrophil (CD16low)
Eosinophil	Eosinophil
Basophil	Basophil
Conventional Dendritic Cell	Conventional Dendritic Cell
Plasmacytoid Dendritic Cell	Plasmacytoid Dendritic Cell

Whole Blood CyTOF marker MSI (BCT)

Cells were filtered as described in the section above, and additionally DNA markers and bead size markers were removed. Features consisted of cell membrane protein markers mean signal intensity (MSI) per cell population. Features with more than 80% missing values were removed, and counts were scaled. Batch correction to correct for the effects of processing batches (phase) and plates was performed with the *removeBatchEffects* function from limma (9).

Clinical data processing

Convalescent symptom groups and longitudinal associations

Symptoms reported at hospital admission (baseline) and during the convalescent period were categorized into three mutually exclusive groups: Cardiopulmonary (dyspnea, cough), systemic (fatigue, myalgia, chills, fever), and other (GI – nausea, vomiting; neurologic – anosmia, headache; upper respiratory – sore throat, red eyes/conjunctivitis). Reported symptoms within each group were counted to identify participants who had reported one of these symptoms during at least one convalescent timepoint, as well as participants who had reported a symptom at two or more timepoints, including baseline.

Acute infection trajectory group definition

Respiratory ordinal scores were assigned to each acute participant visit based on recorded clinical data, such as respiratory supports and hospitalization status (11). Unsupervised clustering of these scores over time was used to group participants into five groups of disease severity (11). The clustering was run twice: once for the initial clinical data analysis and again once all data collection and cleaning was complete, generating two sets of groups. This analysis uses the latter groups, based on the more complete dataset. These groups were classified as: short hospital stay (trajectory 1: n=232; 20%); intermediate hospital stay (trajectory 2: n=265; 23%); intermediate hospital stay with discharge limitations (trajectory 3: n=337; 29%); prolonged hospital stay (trajectory 4: n=222; 19%); and death in acute phase (trajectory 5: n=108; 9%).

Vaccination data collection

Vaccination data was captured in the four convalescent surveys. As enrollment started in March 2021, there was no vaccine data collection built into the acute phase of the study, so data for participants vaccinated prior to the convalescent phase is limited. In the surveys, participants

were asked if they had received a vaccine since the last visit, the brand of vaccine, and the date of vaccination.

Antibody titers

Anti-SARS-CoV-2 spike, and receptor binding domain (RBD) antibodies were measured by enzyme-linked immunosorbent assay (ELISA) in blood serum as previously described (12). Readouts were generated in duplicate for serology measures, endpoint titers, and area under the curve (AUC) values. AUC was chosen as the main readout as it considers not only the endpoint titer of ELISA curves but also the magnitude of the signal at each dilution.

Viral load

SARS-CoV-2 viral load was assessed by RT-PCR of the viral N1, and N2 genes from nasal swab samples in a centralized laboratory as previously described (1). Gene PCR cycle threshold (Ct) values were chosen as the main readout.

MOFA model construction

Multi-Omics Factor analysis is a computational method to identify the principal sources of variation in multi-omics datasets. It employs a variational Bayesian framework, modeling the high-dimensional multi-omics assays as a product of a lower dimensional factor loadings and scores with error. The MOFA factor construction procedure is unsupervised, without knowledge of the outcome variable. In this work, we compared the predictive performance of unsupervised MOFA factors and supervised SPEAR factors. As the SPEAR model construction procedure does not handle missing values, we also employed the MOFA model for missing value imputation prior to constructing the SPEAR model.

We trained separate MOFA models with the MOFA2 (13) R package for the Train and Test cohorts, to avoid data leakage between these sets. Both models were obtained initially specifying 250 factors but dropping factors that explain less than 1% of variance in all assays. After this selection the MOFA model on the Train data comprised 156 factors, and the MOFA model on the Test data comprised 48 factors. The following summed explained variance per assay across all factors was obtained: Train – SO: 56.4%, PPT: 19.2%, PPG:45.2%, PMG:59.2%, PGX: 79.2%, BCT: 70.3%. Test – SO: 39.4%, PPT:12.9%, PPG: 44.9%, PMG: 44.2%, PGX: 74.3%, BCT: 63.6%.

SPEAR model training

Data imputation

Signature-based multiPle-omics intEgration via lAtent factoRs (14) (SPEAR) performs supervised dimensionality reduction to identify predictive low-dimensional factors from high-dimensional multi-omics data. SPEAR requires full-rank matrices for constructing a model, thus missing data imputation was first performed using the Train MOFA model described in the previous section.

SPEAR model training for each response variable

As SPEAR supports multiple categories of response types (e.g. Gaussian, ordinal, multinomial), we trained multiple SPEAR models considering several LC response variables:

- A two-class (binomial) response of MIN (minimal deficits) or LC (Long COVID).
- A continuous (Gaussian) response using the Physical, Cognitive, Mental, Psychosocial Impact, and Dyspnea population-normalized PROMIS scale scores.

All SPEAR models were trained using 10-fold cross-validation on the Train cohort (with balanced classes within the two-class model) to generate 80 factors (identified as a sufficient rank by SPEAR). Optimal SPEAR weight (w) was defined as the weight with the lowest overall cross-validated error, resulting in w=1 for the binomial two-class model and w=0 for all Gaussian models.

SPEAR model selection and evaluation

To evaluate SPEAR model performance on the Train cohort, we trained a separate lasso model using the generated SPEAR factors for each trained model in a 10-fold cross-validation procedure. We first evaluated the predictive performance of the SPEAR multi-omics factors trained on the individual PRO survey scores to reconstruct the score values of unseen data in the training cohort by cross-validation using a lasso regression model (Figure S3B), and the root mean squared error (RMSE) of the predicted PROMIS scores against the true PROMIS scores as a performance metric. The SPEAR Physical model was the best performing, with lowest prediction error, and was selected for further evaluation. We then assessed the performance of the SPEAR Physical factors as well as factors obtained from the SPEAR model trained on the binary LC status (SPEAR LC) to predict binary LC status using a lasso classifier model. Performance was measured in a 10-fold cross-validation procedure on the training set and evaluated via the average Area Under the Receiver Operating Characteristic (AUROC) scores of 100 bootstrapped model training iterations.

Computing factor scores for the Test cohort and acute phase visits immune profiling

SPEAR factor scores for preprocessed testing and acute visit measurements samples were calculated using coefficients derived from the pretrained SPEAR Physical model on the Train cohort.

Statistical analysis and association analysis

Factor scores and geometric mean scores association

Factor scores or geometrical mean scores were associated with LC (MIN vs LC labels) and PRO groups by fitting a linear mixed effect model (*lmer* function from the lme4 R package (15)) with the factor score to associate as response variable and endpoint (MIN/LC, PRO group, trajectory group) as covariate, adjusting for sex, discretized age quantile and visit number as fixed effects and enrollment site and participant as random effects using the following formula: *Score ~ endpoint + discretized age quantile + sex + visit number + (1|enrollment site/participant ID)*. We tested for the overall significance of the endpoint term with a goodness of fit Chi-Square test. Associations are shown for each participant in the extended test cohort unless otherwise indicated, which includes Test cohort participants with and without PROMIS Physical score values as well as participants in the Train cohort with no available PROMIS Physical score for any measurement, and thus not included in the SPEAR Physical model training (Figure S4). Effect sizes, when indicated, were calculated as the estimated difference between the score means of the compared endpoint groups by the lmer model divided by the squared root sum of the variances of all included random effects and residuals, as previously described (16). To assess whether the association was significant after accounting for acute trajectory group, we subset the dataset by visit number and performed the same association, including trajectory group and removing visit number as covariates. P-values were then adjusted across all tests performed on the acute or convalescent visits. To evaluate whether the association was maintained for males and females, we performed

the same association after subsetting the dataset to males and females and removing the "sex" covariate from the model.

Factor scores and geometric mean scores longitudinal association

Factor scores or geometrical mean scores were longitudinally associated with LC (MIN vs LC labels), PRO groups, and acute trajectory groups by fitting a smoothing splines generalized additive mixed model (gamm) from the *gamm4* R package with the factor score or analyte to associate as response variable and days after admission and endpoint (MIN/LC label, PRO group, or trajectory group) as covariates, adjusting for sex and discretized age as fixed effects and enrollment site and participant ID as random effects using the following formula: *Score ~ endpoint + discretized age quantile + sex + s(event date, bs = "ts") + s(event date, bs = "ts", by = endpoint) + (1|enrollment site/participant ID)*. We tested for the overall significance of the endpoint term with a goodness of fit Chi-Square test. Associations are shown for each participant and visit in the extended test cohort unless otherwise indicated, which includes Test cohort participants with and without PROMIS Physical score values as well as participants in the Train cohort with no available PROMIS Physical score values for any measurement, and thus not included in the SPEAR Physical model training. To evaluate whether the recovery factor was able to discriminate between MIN vs LC within participants with the same acute trajectory group, we performed the same longitudinal association after subsetting by trajectory group (Figure S5C).

Blood CyTOF cell frequencies longitudinal association

Blood CyTOF cell frequencies were associated with the recovery factor by fitting a linear mixed effect model (lme function from the nlme R package) with the factor score as the response variable and individual cell subset frequency as a covariate, adjusting for sex and discretized age as fixed effects and enrollment site and participant ID as random effects using the following formula: *Score ~ cell frequency + sex + discretized age quantile, random = ~1|enrollment site/participant ID*. We tested for the significance of the cell subset frequency with a Wald test. The acute and convalescent cell type associations used the combined train and test splits for those time points. The model coefficient of each cell type was quantile normalized for comparison.

Effect of vaccination on factor scores

To assess the effect of vaccination on the factor scores, we fit a linear mixed effect model (*lmer* function from the lme4 R package (15)) with the factor score as the response variable and endpoint (vaccinated yes/no, within 3 weeks after vaccine dose yes/no) as a covariate, adjusting for sex, discretized age quantile and visit number as fixed effects and enrollment site and participant as random effects using the following formula: *Score ~ endpoint + discretized age quantile + sex + visit number + (1|enrollment site/participant ID).* We tested for the overall significance of the endpoint term with a goodness of fit Chi-Square test.

Factor annotation and enrichment analysis

Gene Set Enrichment Analysis (GSEA) of the multi-omics factors was performed as previously described (8). Briefly, the KEGG, Molecular Signatures Database (MSigDB), and Subpathway publicly available knowledgebases were used for functional enrichment.

- KEGG (17): KEGG pathways and their corresponding gene and metabolite mappings were extracted using the KEGG REST API (Release 102.0), and were used for SO, PMG, PPT, PPG and PGX assay annotation.

- Hallmark (18): Hallmark pathways were extracted from the MSigDB database (Homo sapiens) using msigdbr (version 7.5.1), and were used for SO, PPT, PPG and PGX assay annotation.
- Subpathway: the subpathway database provided by Metabolon was used for metabolite annotation (PMG assay) (19–21).

GSEA was conducted on each of the multi-omics assays based on the SPEAR Physical model's Factor loadings, which are a measure of the model's ranking of relative importance for each feature for predicting the PROMIS Physical score (response variable). The clusterProfiler (22) R package was used for GSEA computation. We then computed a multi-omics joint p-value from assays which contained non-zero features in each pathway using the Cauchy combination test (23), which is robust to the dependence structure of the underlying p-values to be combined.

Geometric mean score

Geometric mean scores were calculated as previously described (24). First, per-sample scores were calculated from log-transformed analyte expression values by taking the difference between the geometric mean of positive signature analyte values and the geometric mean of negative signature analyte values. The "Hallmark Heme Metabolism" and "Androgenic Steroids" signatures consisted of the leading edge features from the corresponding recovery factor enrichment analysis results. The "Significant SPEAR Analytes" signature was constructed using all multi-omics analytes identified as significantly contributing to the recovery factor, indicated by a SPEAR-assigned posterior probability >= 0.95 (rounded to two significant figures). Finally, the "Combined" signature used the union of the analytes from "Hallmark Heme Metabolism", "Androgenic Steroids", and "Significant SPEAR Analytes" signatures, conserving analyte directionality across signatures.

Heme Metabolism validation in the Hanson et al. (2024) and Karisola et al. (2025) cohorts

We accessed publicly available gene counts and LC symptom group designations from a non-hospitalized COVID-19 cohort published by Karisola et al (25) and a mixed non-hospitalized and hospitalized cohort published by Hanson et al (26). For each cohort, gene counts were CPM-normalized using the *cpm* function in edgeR (27). Participant age was discretized into 5 quantiles for consistency across statistical analyses. Geometric means were calculated using the same set of leading edge genes for Hallmark Heme Metabolism as described in the previous section. The Hanson et al. analysis included 38 participants with persisting symptoms (PS) and 49 participants with no persisting symptoms (NPS), with timepoints from 0-90 days post symptom onset. Association with LC symptom groups was determined by fitting a linear mixed effect model with the geometric mean as response variable and PASC symptom groups as predictor, adjusting for sex, discretized age, and time bin (customized range of dates from first report of COVID-19 symptoms) as fixed effects and participant ID as a random effect using the following formula: *Score ~ endpoint (PS vs. NPS) + discretized age quantile + sex + time bin + (1| participant ID).* Significance of the LC symptom group term was determined with a goodness of fit Chi-Square test. The Karisola et al. analysis included 50 participants with LC and 57 recovered participants, none of whom were hospitalized for COVID-19. Association with LC symptom groups was determined by fitting a linear model with the geometric mean as response variable and LC symptom groups as predictor, adjusting for sex and discretized age using the following formula:

Score ~ endpoint (LC vs. Recovered) + discretized age quantile + sex. Significance of the LC symptom group term was determined with a goodness of fit Chi-square test.

Machine learning approach to compare recovery factor and clinical models

SPEAR acute and convalescent models

Lasso classification models to classify participants according to the LC status (LC vs MIN) were constructed using the SPEAR factor scores for each participant at each of the visits (SPEAR Visit 1-10) on the Train cohort. Additionally, models including the average of the SPEAR factor scores at the acute phase (SPEAR acute) or at the convalescent phase (SPEAR conv) were compared. Whenever specified, the clinical features from the clinical models described below were included during model training.

SPEAR significant analytes model

To compare the performance of the SPEAR models utilizing the factor scores models derived from the full analyte model to the set of SPEAR significant analytes, we constructed a lasso classification model to classify participants according to the LC status utilizing the average values of the 26 SPEAR significant analytes (analytes with SPEAR posterior probability >= 0.95) during the convalescent phase.

Clinical model construction

A lasso classification model using baseline clinical measurements, denoted as the clinical model, was constructed on the Train Cohort to classify participants according to the LC status for comparison with the SPEAR acute and convalescent models. The clinical models included the following features: age at enrollment, sex, body mass index (BMI), length of hospital stay, Sequential Organ Failure Assessment (SOFA) score, mean values of Spike IgG levels and viral load (N1 Ct) during the acute phase, and the presence of comorbidities including hypertension, diabetes, chronic cardiac disease, chronic kidney disease, malignant neoplasms, chronic neurological disorders, liver disfunction/failure, history of transplants, smoking/vaping, asthma, respiratory diseases other than asthma, substance use, HIV infection, and the total number of comorbidities.

Model training, evaluation, and performance comparison

All the models mentioned above were evaluated on a 10-fold cross-validation setting on the Train Cohort. Bootstrapping of N=100 models trained updating the random seed for the fold creation was utilized to compare the average area under the ROC curve (AUROC) performance on the cross-validation folds. The average AUROC values across bootstrapped samples were used for plotting and computing the statistical significance of performance differences across models, employing a t-test, and adjusting the p-values when relevant with the Benjamini-Hochberg approach.

SUPPLEMENTAL FIGURES

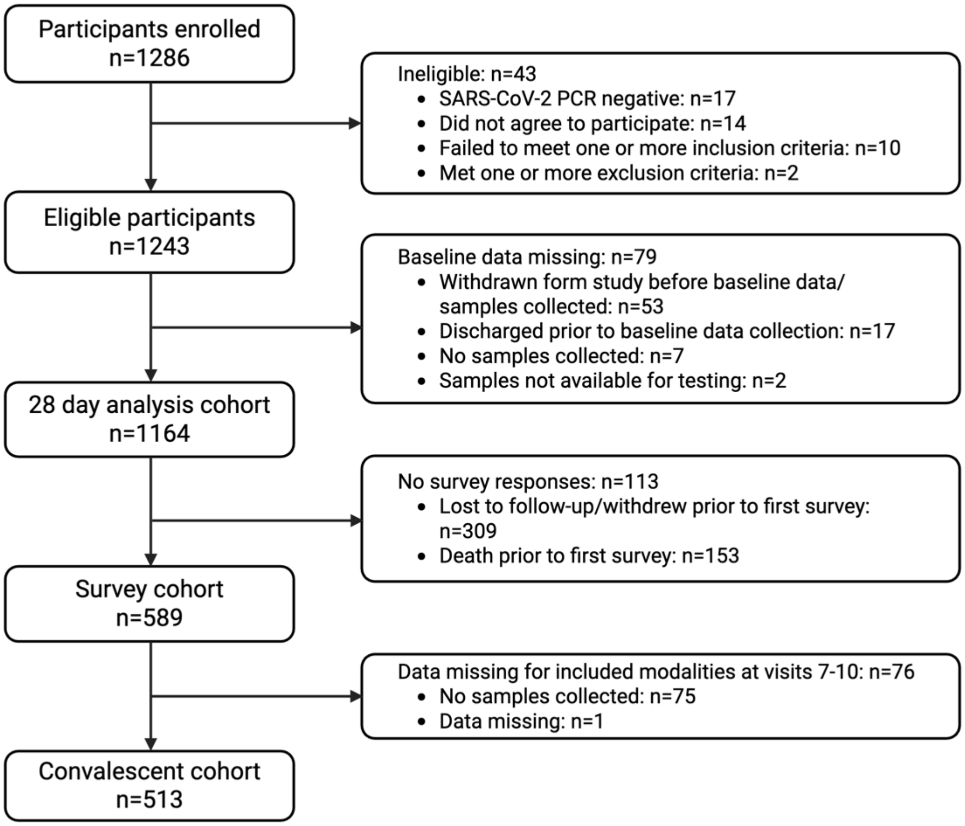

Figure S1. IMPACC Convalescent Cohort STROBE diagram.

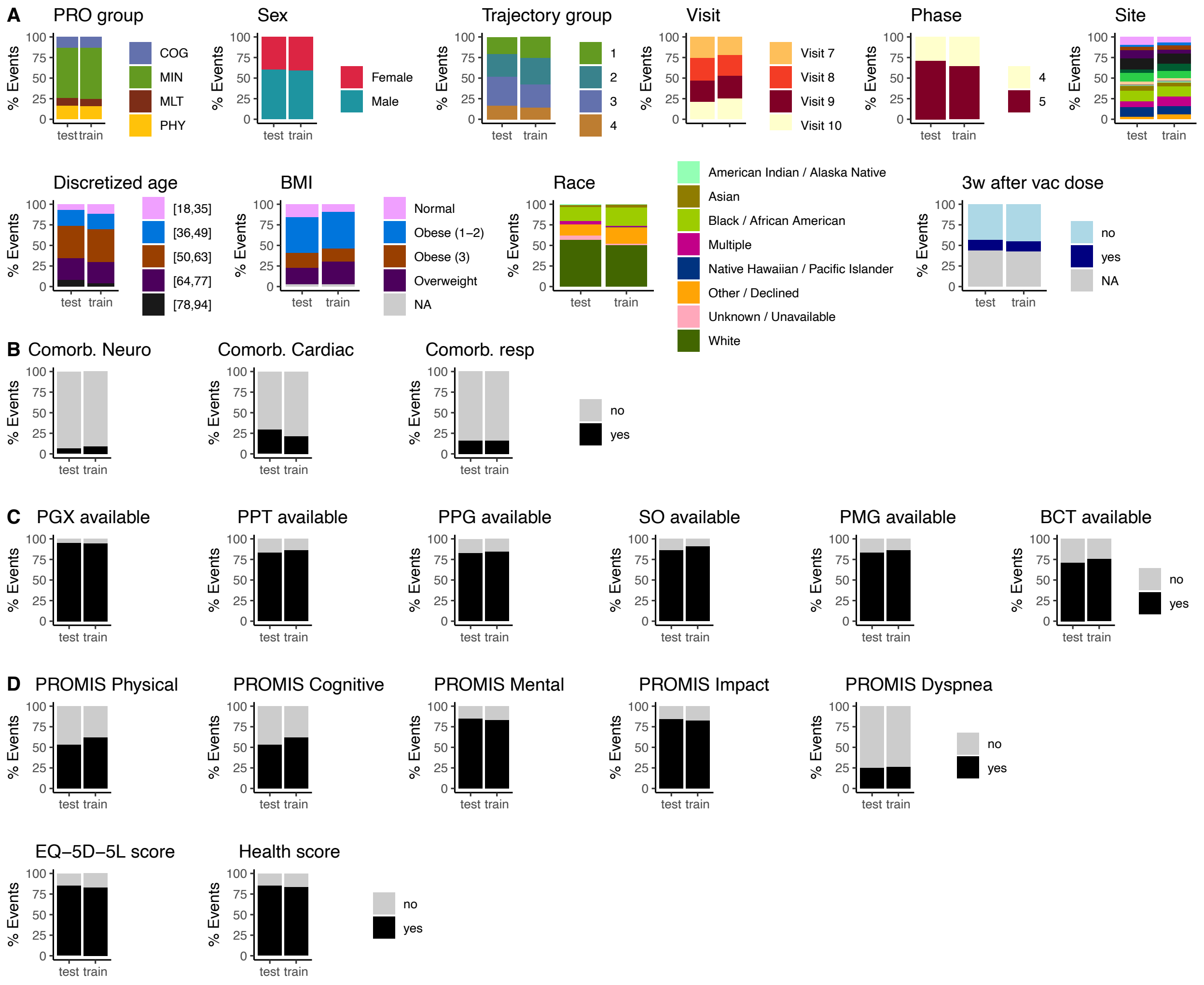

Figure S2. IMPACC convalescent train and test cohort split evaluation. (A) Balance of clinical characteristics within the Train and Test cohorts events including PRO group, sex (physician determined or reported sex at birth), trajectory group (acute COVID-19 disease severity measure), visit (target point of sample collection), phase (sample collection batch), enrollment site, discretized admit age, body mass index (BMI) and whether the sample was collected 3 weeks after a reported vaccine dose (3w after vac dose). **(B)** Balance of comorbidities (neurological, cardiac and respiratory) among participants in the train and test cohorts. **(C)** Availability of immunophenotyping assay data among participants in the train and test cohorts. **(D)** Availability of PROMIS scale scores among participants in the train and test cohorts.

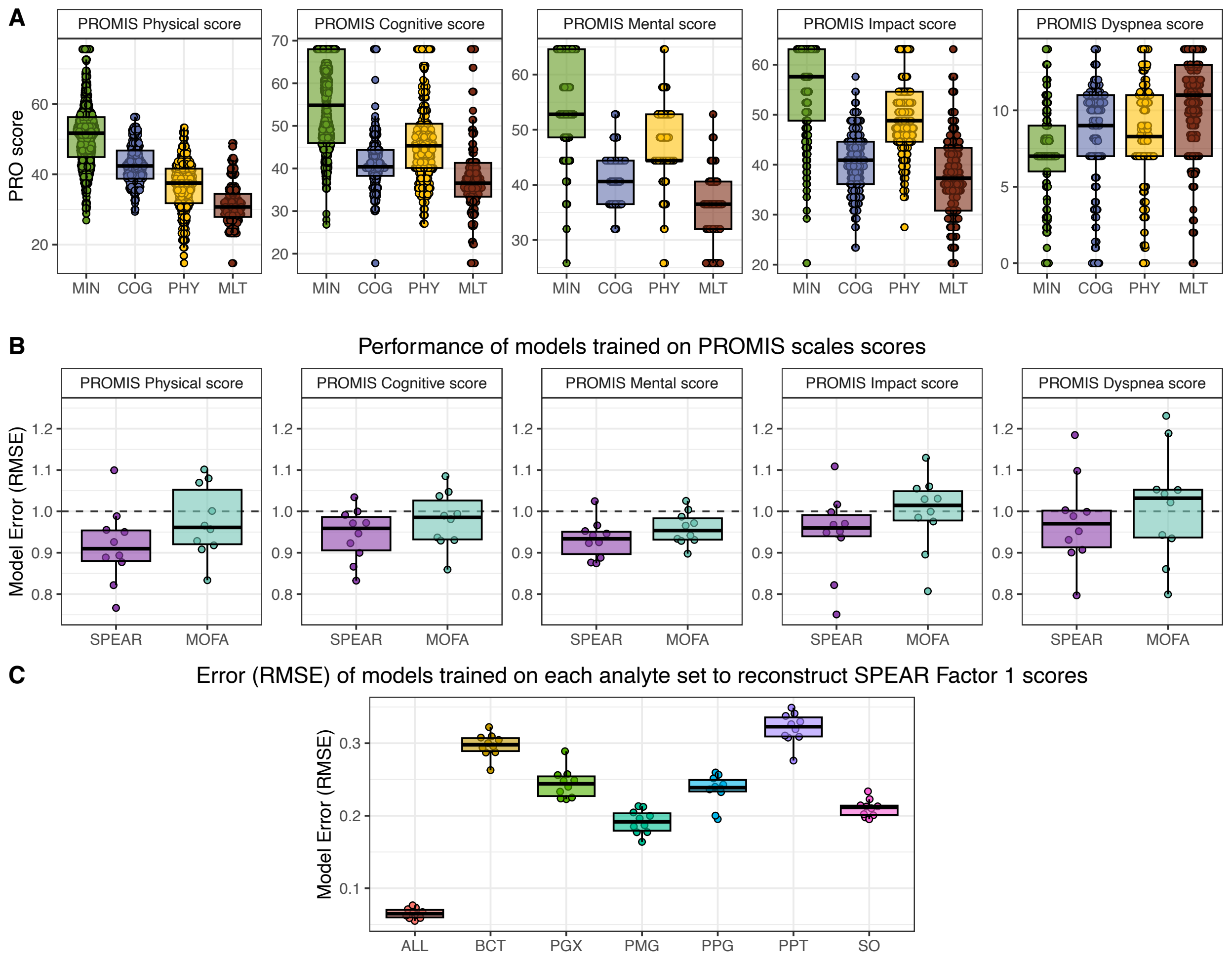

Figure S3. Evaluation of SPEAR and MOFA models trained on PROMIS scale scores. (A) PROMIS scale scores values for each PRO group. **(B)** Model error evaluated as root mean squared error (RMSE) of lasso regression models trained on SPEAR and MOFA factors using 10-fold cross-validation on the train cohort with each of the PROMIS scale scores as response variable (lower RMSE values indicate a better model). Note that lower model error indicates better model performance. **(C)** Model error (RMSE) of lasso regression models trained on each individual analyte layer and all analytes (ALL) to reconstruct SPEAR Factor 1 scores. Individual analyte layers include Blood CyTOF MSI values (BCT), PBMC transcriptomics (PGX), plasma metabolomics global (PMG), plasma proteomics global (PPG), plasma proteomics targeted (PPT) and serum O-link (SO).

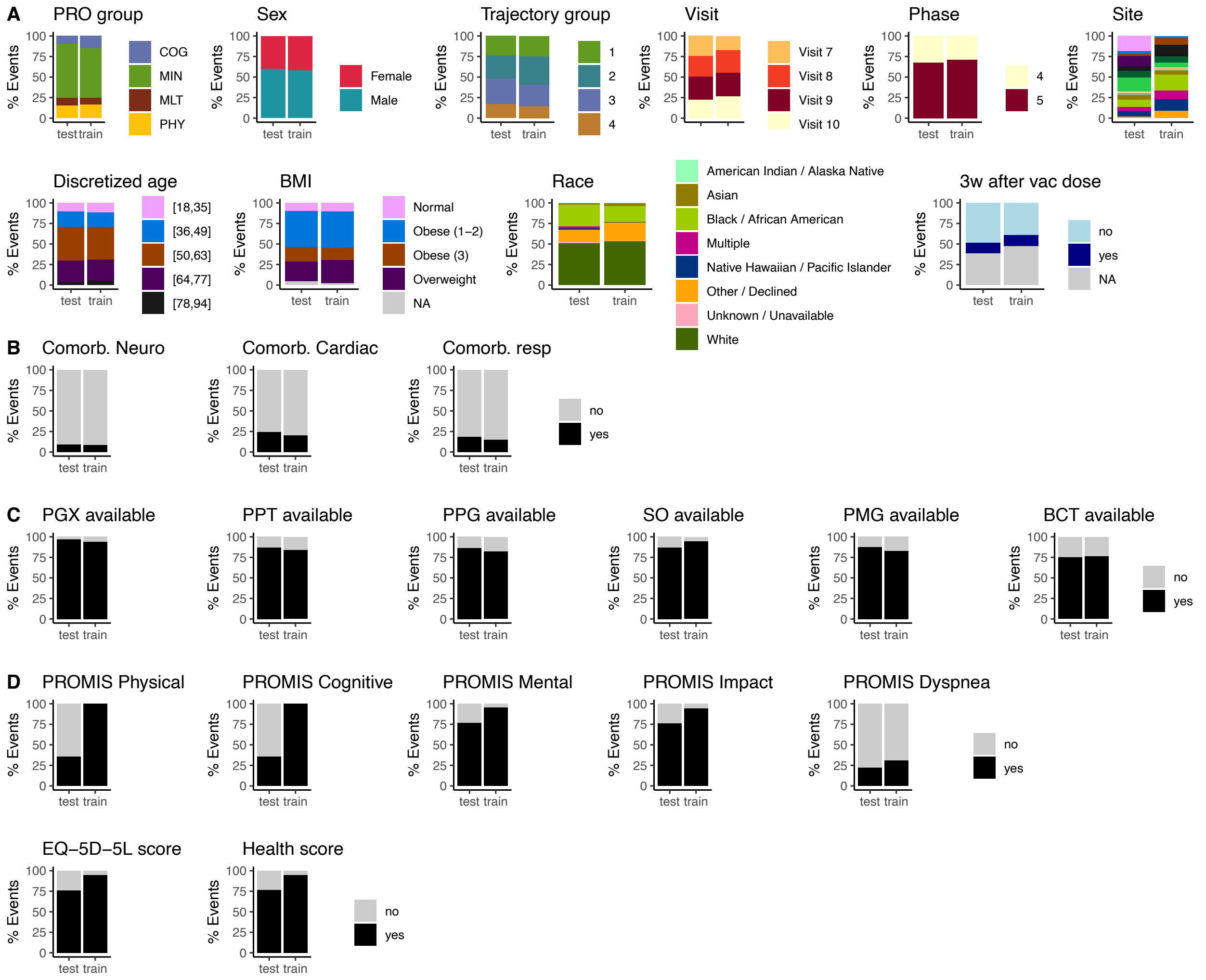

Figure S4. IMPACC convalescent train and extended test set split evaluation. To associate the recovery factor with LC vs MIN participants or PRO groups we considered an extended test set which comprised all participants in the Test cohort (with and without PROMIS Physical score) and participants in the Train cohort which did not have a PROMIS Physical score available for any of their measured samples and thus were not included in model training. The balance of the clinical characteristics **(A)**, comorbidities **(B)**, immunophenotyping assay data availability **(C)** and PRO survey score availability **(D)** of these two sets is shown.

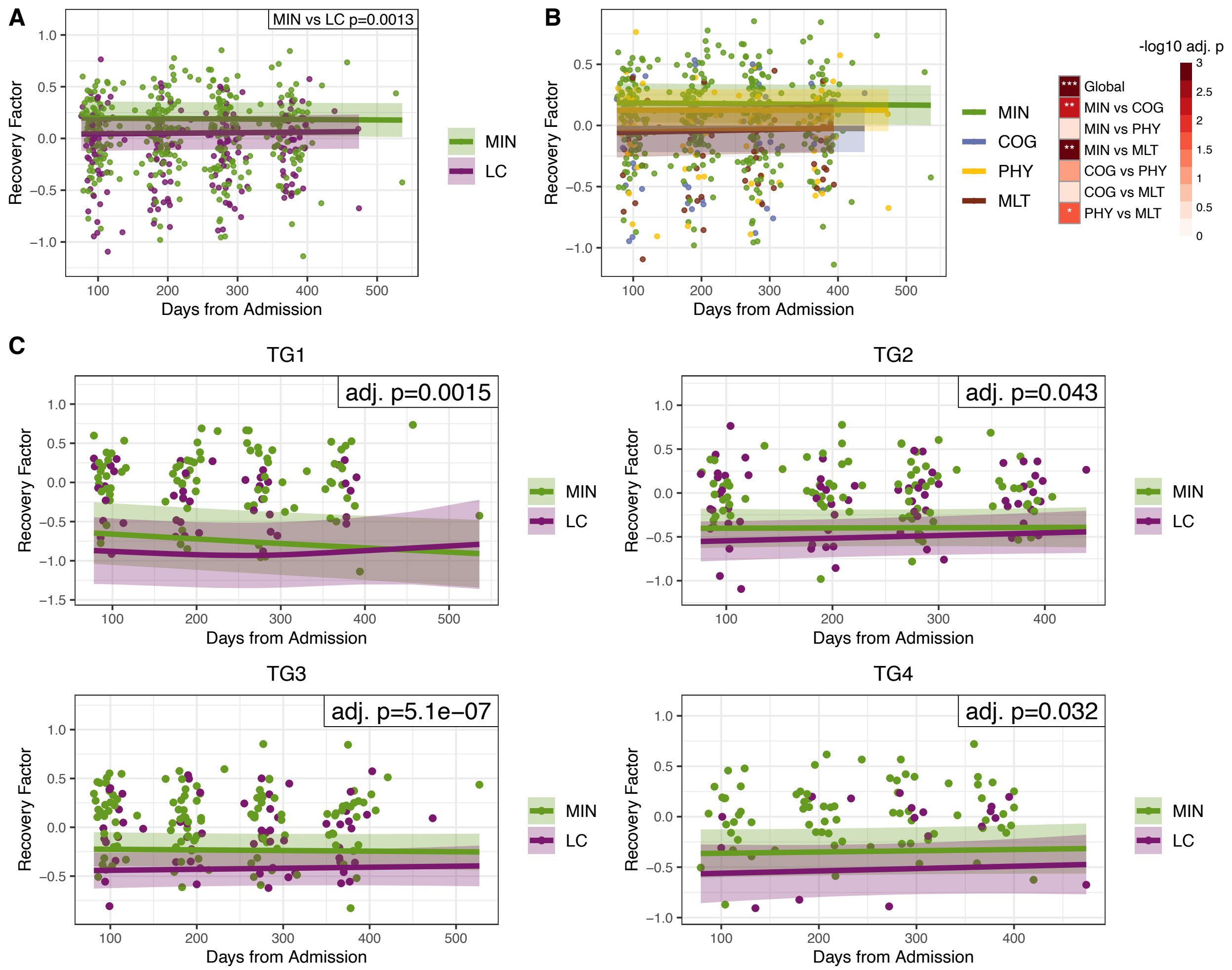

Figure S5. Recovery factor is longitudinally associated with long COVID. (A) Recovery factor scores for extended Test set MIN and LC participants. The p-value shows the longitudinal association of the recovery factor scores with MIN vs LC. **(B)** Recovery factor scores for the test cohort participants for each PRO group (MIN, COG, PHY, MLT). The heatmap shows the longitudinal association of the recovery factor with the PRO groups when considering global differences across all groups (Global) and pairwise differences among PRO groups. **(C)** Recovery factor scores and their association with MIN vs LC, subset by acute trajectory group.

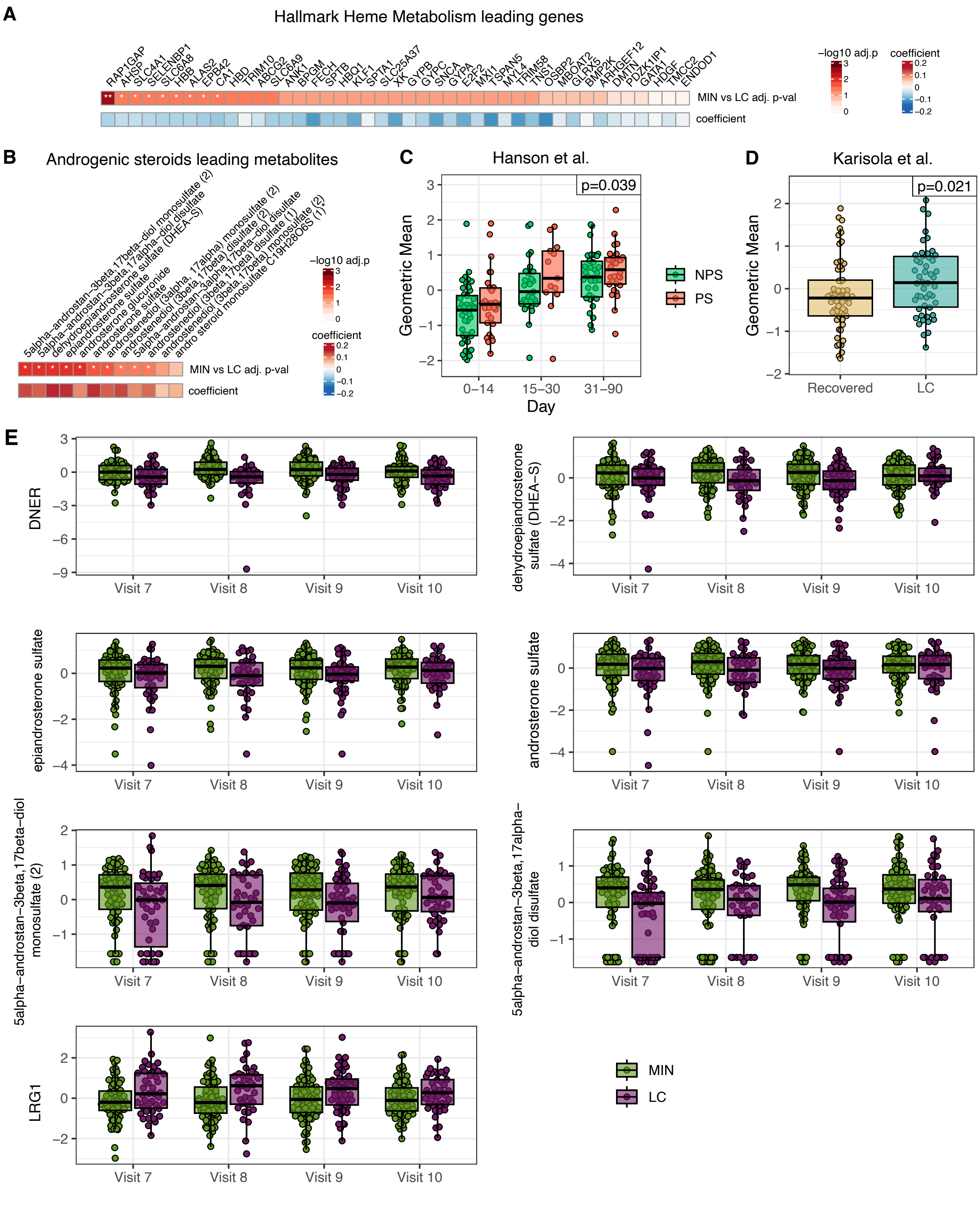

Figure S6. Individual associations of analytes from pathways associated with the recovery factor or significant Spear analytes with MIN vs LC status, and evaluation of the recovery factor Hallmark heme metabolism signature in an external cohort. (A) Longitudinal association of the Hallmark heme metabolism pathway set leading genes with MIN vs LC (top;

adj. intercept p-value), and SPEAR factor loadings (bottom; coefficient in the factor). **(B)** Longitudinal association of the Androgenic steroids set leading metabolites with MIN vs LC (top; adj. intercept p-value), and SPEAR factor loadings (bottom; coefficient in the factor). For panels A-B, (* adj. p-value < 0.05, ** adj. p-value < 0.01). (**C**) The Hallmark heme metabolism geometric mean signature was evaluated in whole blood transcriptomics measurements from participants with persisting symptoms (PS, N=38) and no persisting symptoms (NPS, N=49) in the Hanson et al. (2024) (26) cohort. (**D**) The recovery factor Hallmark heme metabolism geometric mean signature was evaluated in PBMC transcriptomics measurements from participants with LC (N=50) and recovered (N=57) participants from the Karisola et al. (2025) (25) cohort. (**E**) Significant SPEAR analyte values for the Test cohort in the MIN vs LC groups at the convalescent phase visits. Plots are shown only for Significant SPEAR analytes that were also significantly associated with MIN vs LC status (Figure 3B).

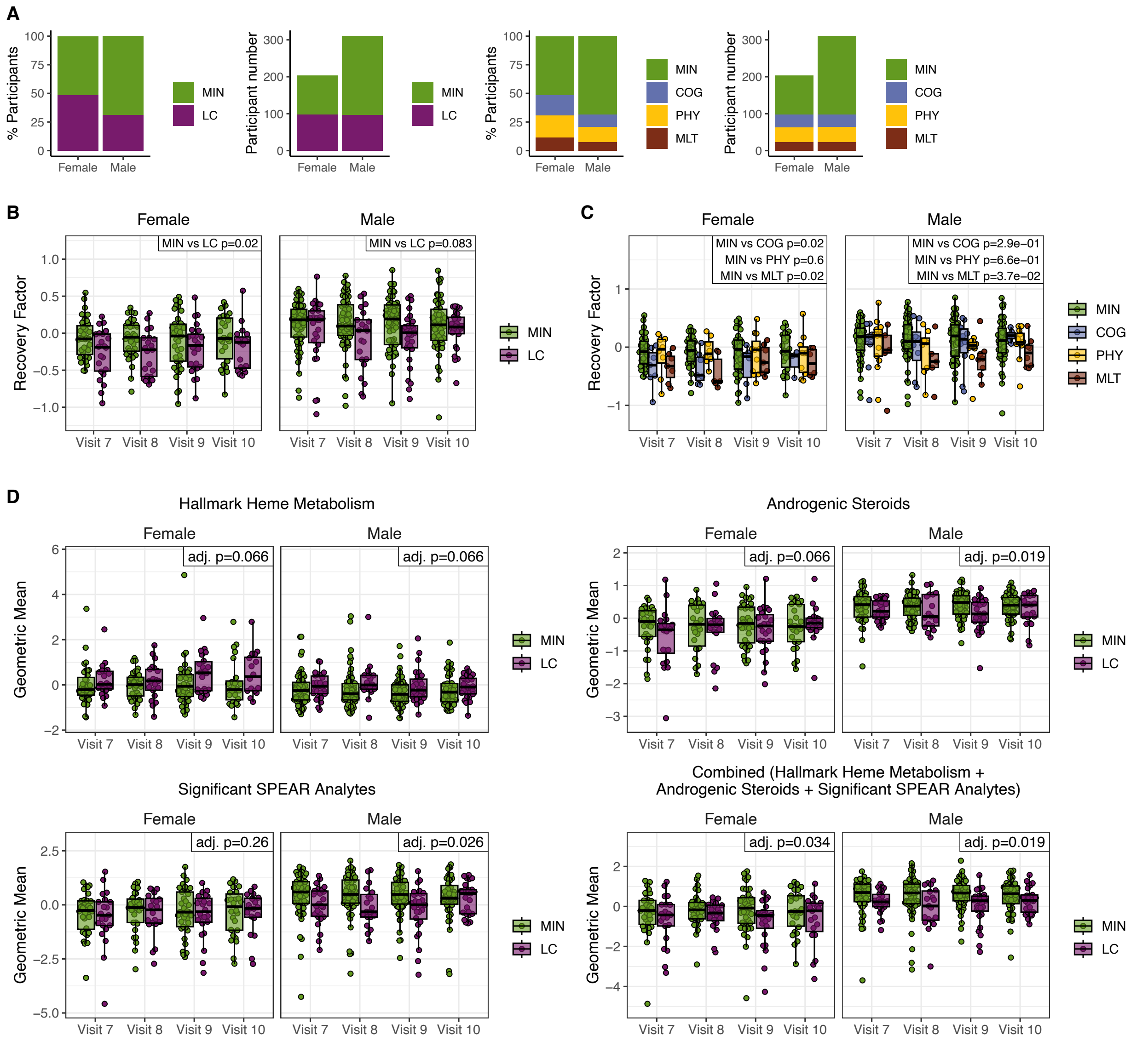

Figure S7. Effects of sex on the recovery factor. (A) Percentage and number participants in the MIN and LC group as well as individual PRO groups for Females and Males in the Convalescent Cohort. **(B)** Recovery factor scores for the Test cohort for MIN and LC groups stratified by sex. **(C)** Recovery factor scores for the Test cohort for PRO groups stratified by sex. **(D)** Test cohort geometric mean scores stratified by sex. Adjusted p-values show the association of the recovery scores with MIN vs LC. Associations were performed separately for males and females for each score.

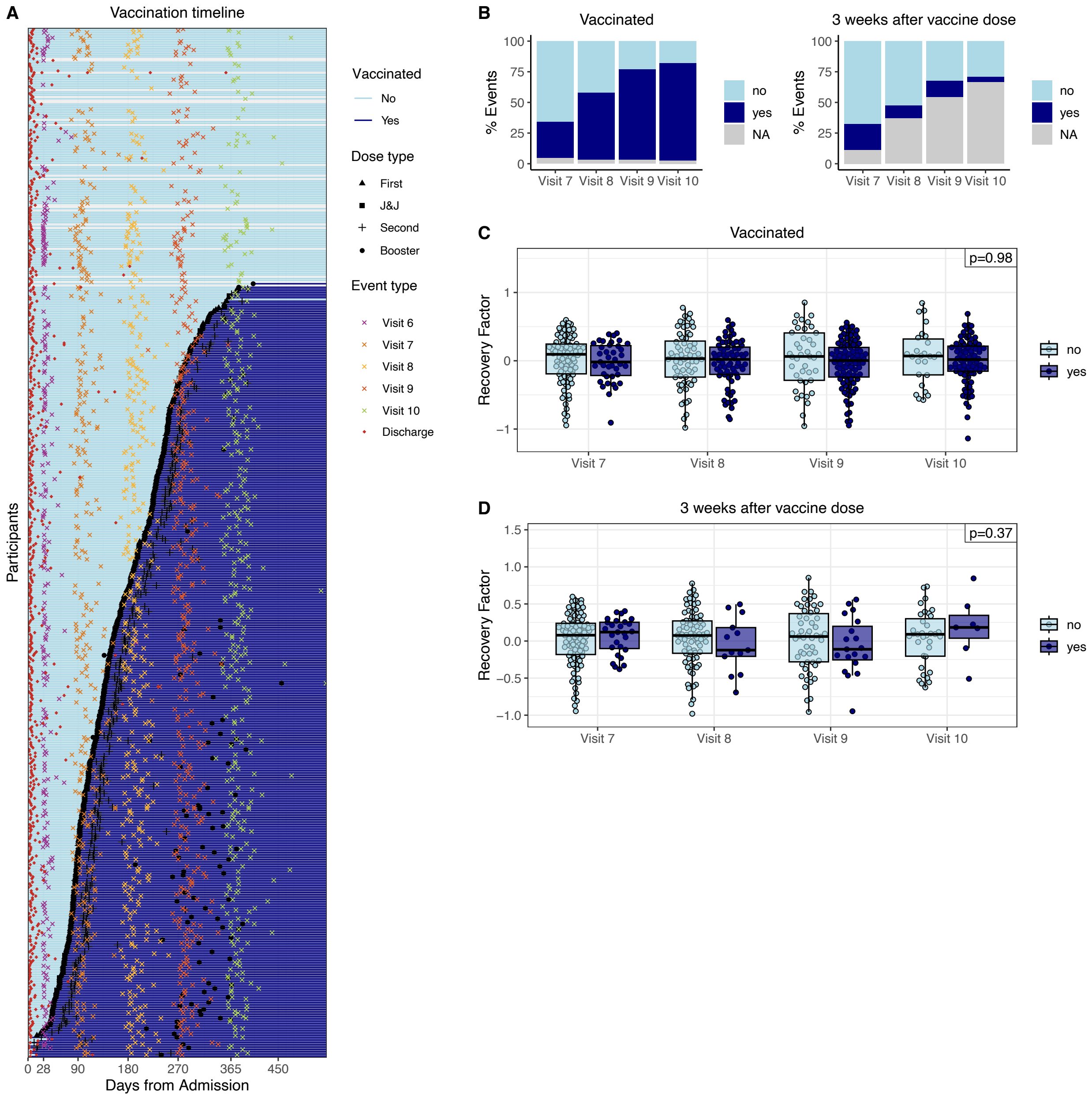

Figure S8. Effects of vaccination on the recovery factor. (A) Outpatient visits, hospital discharge and SARS-CoV-2 vaccination events timeline in days from admission for each participant in the Convalescent Cohort. Participants were ordered according to their first vaccination dose. **(B)** Percentage of events in each visit occurring after the participant received the first vaccine dose (vaccinated) or within 3 weeks after any vaccine dose. NA indicate events where the participant vaccination status, or exact vaccination date is unknown. **(C)** Recovery factor scores for events occurring before and after a participant received their first vaccine dose. **(D)** Recovery factor scores for events occurring within 3 weeks after any vaccine dose. P-values indicate the association of the recovery factor scores with the vaccination status.

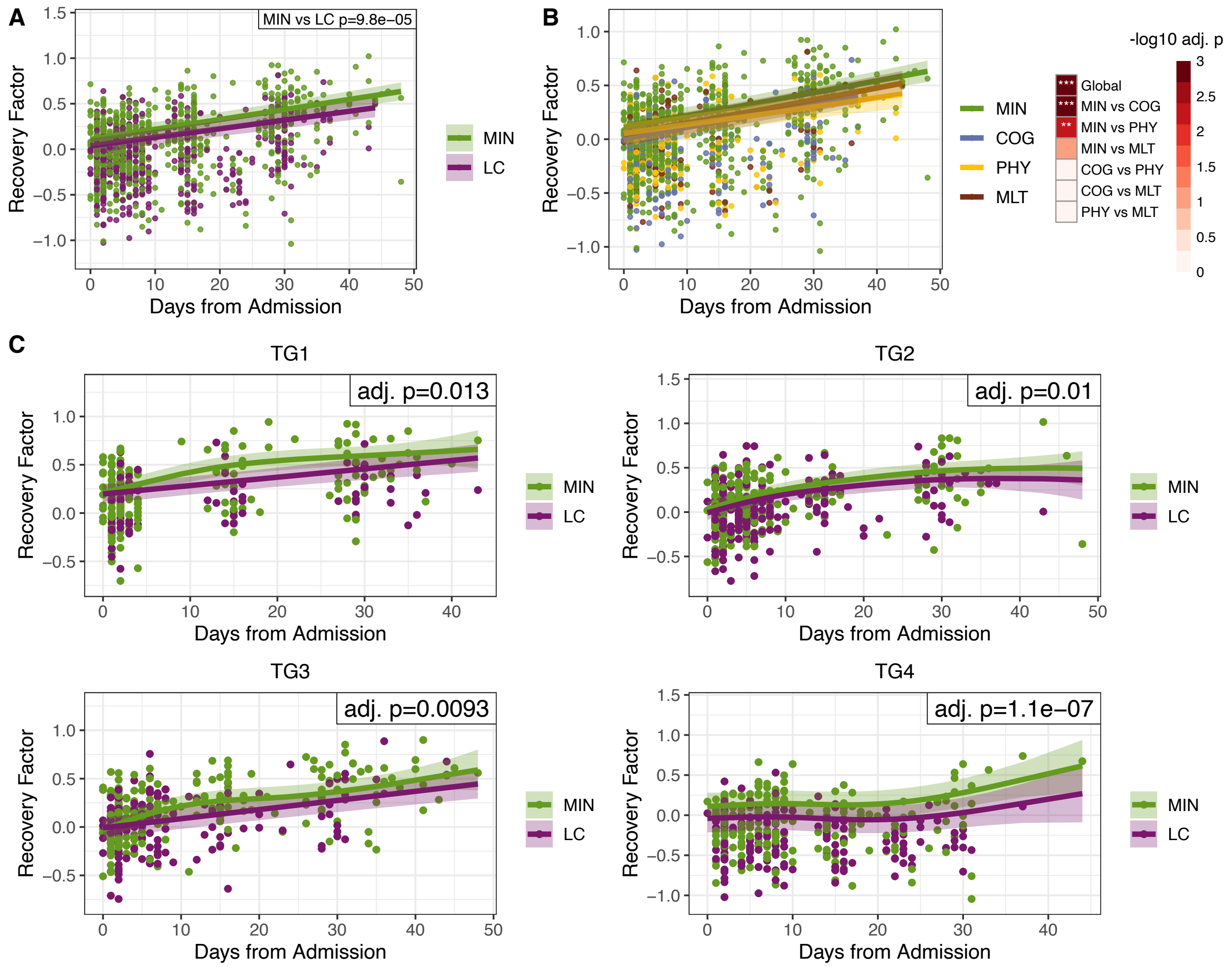

Figure S9. Recovery factor is longitudinally associated with long COVID already during the acute phase. (A) Recovery factor scores for MIN and LC participants during the acute disease phase. **(B)** Recovery factor scores for PRO group participants during the acute disease phase. The heatmap shows the longitudinal association of the recovery factor with the PRO groups when considering global differences across all groups (ALL PRO) and pairwise differences among PRO groups (*** adj. p < 0.001, ** adj. p < 0.01). **(C)** Recovery factor scores during the acute phase and their association with MIN vs LC, subset by acute trajectory group. Adjusted p-values indicate the association of the recovery factor scores with MIN vs LC.

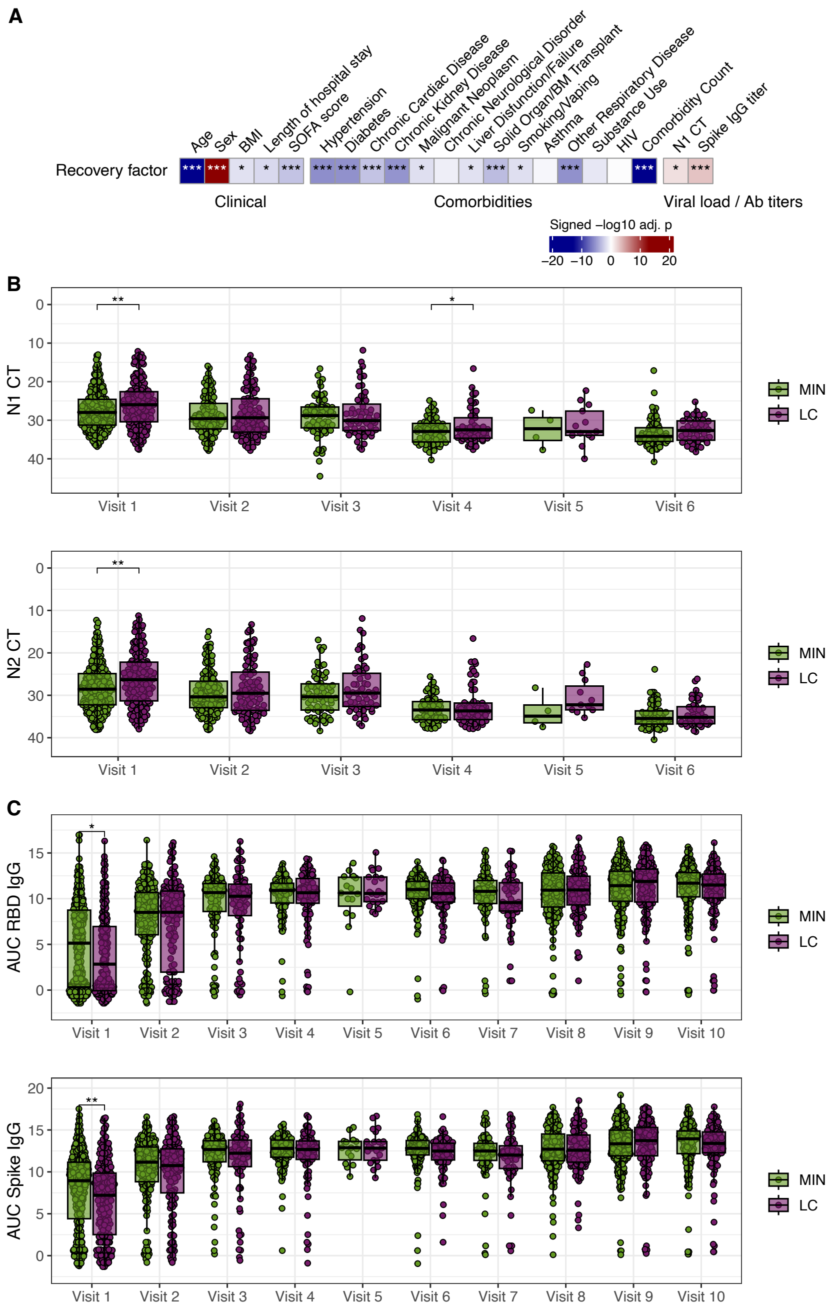

Figure S10. Clinical features included in machine learning models. (A) The heatmap shows signed adjusted p-values indicating the clinical feature term significance from a linear mixed-effect model with enrollment site and participant as random effects to explain the convalescent phase recovery factor scores. Sex and discretized age were further adjusted as fixed effects for clinical features other than sex and age. Color represents the term directionality. The Sex variable test shows male sex association. **(B)** SARS-CoV-2 N1 and N2 gene PCR cycle threshold (Ct) values indicating viral load for the MIN and LC participant groups. Lower Ct values indicate higher viral loads, so the y axis is reversed. **(C)** Antibody titers against the SARS-CoV-2 virus full Spike protein (Spike IgG) and Receptor Binding Domain (RBD IgG). Area under the curve (AUC) values for the MIN and LC participant groups are shown.

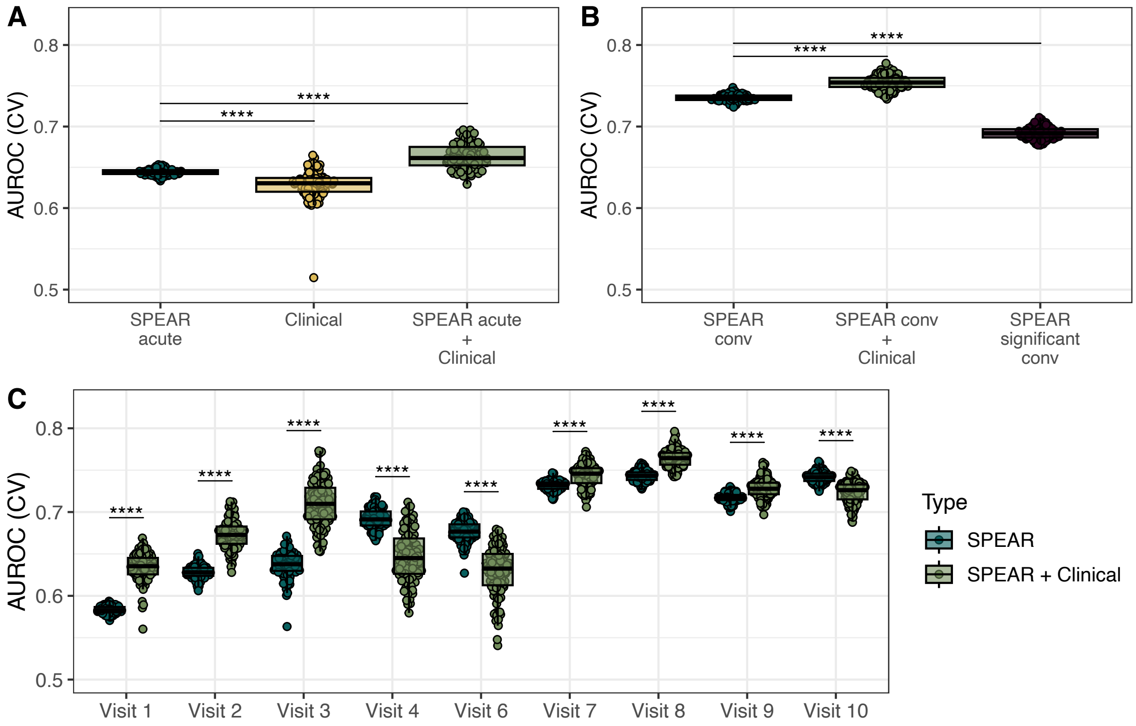

Figure S11. Machine learning models based on the recovery factor scores at the acute and the convalescent phase together with clinical features predict LC status during the convalescent phase. (A) Predictive performance of lasso classification models trained on the average of the recovery factor scores in the acute phase (SPEAR acute, mean cross-validation AUROC 0.64), the clinical variables shown in A (Clinical, mean cross-validation AUROC 0.63), as well as the combination of the recovery factor scores in the acute phase and clinical features (SPEAR acute + Clinical, mean cross-validation AUROC 0.66). **(B)** Predictive performance of lasso classification models trained on the average of the recovery factor scores in the convalescent phase (SPEAR conv, mean cross-validation AUROC 0.74), the combination of the recovery factor scores in the convalescent phase and the clinical variables shown in A (Spear conv + Clinical, mean cross-validation AUROC 0.75), and a model trained on the average values over the convalescent visits of the 26 SPEAR significant analytes (SPEAR significant conv, mean cross-validation AUROC 0.76). **(C)** Predictive performance of lasso models trained on the recovery factor scores at each of the acute (1-6) and convalescent (7-10) visits, compared to the same models including the clinical features in A. Visit 5 was omitted as there were insufficient measurements for the prediction evaluation. For panels B-D the mean AUROC of a 10-fold cross-validation on the Train Cohort, for 100 bootstrapped model training repetitions are shown. Significance was calculated by standard normal approximation of bootstrapped differences between models (t-test, ****adj. p-value ≤ 0.0001).

CONFLICT OF INTEREST

The Icahn School of Medicine at Mount Sinai has filed patent applications relating to SARS-CoV-2 serological assays, NDV-based SARS-CoV-2 vaccines influenza virus vaccines and influenza virus therapeutics which lists Florian Krammer as co-inventor. Mount Sinai has spun out a company, Kantaro, to market serological tests for SARS-CoV-2 and another company, Castlevax, to develop SARS-CoV-2 vaccines. Florian Krammer is co-founder and scientific advisory board member of Castlevax. Florian Krammer has consulted for Merck, Curevac, Seqirus, GSK and Pfizer and is currently consulting for 3rd Rock Ventures, Sanofi, Gritstone and Avimex. The Krammer laboratory is also collaborating with Dynavax on influenza vaccine development and with VIR on influenza virus therapeutics development. Viviana Simon is a co-inventor on a patent filed relating to SARS-CoV-2 serological assays (the "Serology Assays"). Ofer Levy is a named inventor on patents held by Boston Children’s Hospital relating to vaccine adjuvants and human in vitro platforms that model vaccine action. His laboratory has received research support from GlaxoSmithKline (GSK) and Pfizer, and he is a co-founder of and advisor to Ovax, Inc that develops opioid vaccines. Charles Cairns serves as a consultant to bioMerieux and is funded by a grant from Bill & Melinda Gates Foundation. James A Overton is a consultant at Knocean Inc. Jessica Lasky-Su serves as a scientific advisor of Precion Inc. Scott R. Hutton, Greg Michelloti and Kari Wong are employees of Metabolon Inc. Vicki Seyfer- Margolis is a current employee of MyOwnMed. Nadine Rouphael reports grants or contracts with Merck, Sanofi, Pfizer, Vaccine Company and Immorna, and has participated on data safety monitoring boards for Moderna, Sanofi, Seqirus, Pfizer, EMMES, ICON, BARDA, and CyanVan, Imunon Micron. N.R. has also received support for meetings/travel from Sanofi and Moderna and honoraria from Virology Education and Krog consulting. Chris Cotsapas is a current employee of Vesalius Therapeutics. Adeeb Rahman is a current employee of Immunai Inc. Steven Kleinstein is a consultant related to ImmPort data repository for Peraton. Nathan Grabaugh is a consultant for Tempus Labs and the National Basketball Association. Akiko Iwasaki is a consultant for 4BIO, Blue Willow Biologics, Revelar Biotherapeutics, RIGImmune, Xanadu Bio, Paratus Sciences. Monika Kraft receives research funds paid to her institution from NIH, ALA; Sanofi, Astra-Zeneca for work in asthma, serves as a consultant for Astra-Zeneca, Sanofi, Chiesi, GSK for severe asthma; is a co-founder and CMO for RaeSedo, Inc, a company created to develop peptidomimetics for treatment of inflammatory lung disease. Esther Melamed received research funding from Babson Diagnostics and honorarium from Multiple Sclerosis Association of America and has served on the advisory boards of Genentech, Horizon, Teva, and Viela Bio. Carolyn Calfee receives research funding from NIH, FDA, DOD, Roche-Genentech and Quantum Leap Healthcare Collaborative as well as consulting services for Janssen, Vasomune, Gen1e Life Sciences, NGMBio, and Cellenkos. Wade Schulz was an investigator for a research agreement, through Yale University, from the Shenzhen Center for Health Information for work to advance intelligent disease prevention and health promotion; collaborates with the National Center for Cardiovascular Diseases in Beijing; is a technical consultant to Hugo Health, a personal health information platform; cofounder of Refactor Health, an AI-augmented data management platform for health care; and has received grants from Merck and Regeneron Pharmaceutical for research related to COVID-19. Grace A McComsey received research grants from Rehdhill, Cognivue, Pfizer, and Genentech, and served as a research consultant for Gilead, Merck, Viiv/GSK, and Janssen. Linda N. Geng received

research funding paid to her institution from Pfizer, Inc. Catherine Hough receives support through her institution from the NIH and CDC. David Hafler has received research funding from Bristol-Myers Squibb, Novartis, Sanofi, and Genentech. He has been a consultant for Bayer Pharmaceuticals, Repertoire Inc, Bristol Myers Squibb, Compass Therapeutics, EMD Serono, Genentech, Novartis Pharmaceuticals, and Sanofi Genzyme.

SUPLEMENTAL ACKNOWLEGMENTS

The members of the IMPACC Network are:

National Institute of Allergy and Infectious Diseases, National Institute of Health, Bethesda, MD 20814, USA:
Patrice M. Becker, Alison D. Augustine, Steven M. Holland, Lindsey B. Rosen, Serena Lee, Tatyana Vaysman

Clinical and Data Coordinating Center (CDCC), Precision Vaccines Program, Boston Children's Hospital, Harvard Medical School, Boston, MA 02115, USA:
Al Ozonoff, Joann Diray-Arce, Jing Chen, Alvin T. Kho, Carly E. Milliren, Annmarie Hoch, Ana C. Chang, Kerry McEnaney, Caitlin Syphurs, Brenda Barton, Claudia Lentucci, Maimouna D. Murphy, Mehmet Saluvan, Tanzia Shaheen, Shanshan Liu, Marisa Albert, Arash Nemati Hayati, Robert Bryant, James Abraham, Mitchell Cooney, Meagan Karoly

Benaroya Research Institute, University of Washington, Seattle, WA 98101, USA:
Matthew C. Altman, Naresh Doni Jayavelu, Scott Presnell, Bernard Kohr, Tomasz Jancsyk, Azlann Arnett

La Jolla Institute for Immunology, La Jolla, CA 92037, USA:
Bjoern Peters, James A. Overton, Randi Vita, Kerstin Westendorf

Knocean Inc. Toronto, ON M6P 2T3, Canada:
James A. Overton

Precision Vaccines Program, Boston Children's Hospital, Harvard Medical School, Boston, MA 02115, USA:
Ofer Levy, Hanno Steen, Patrick van Zalm, Benoit Fatou, Kinga K. Smolen, Arthur Viode, Simon van Haren, Meenakshi Jha, David Stevenson, Sanya Thomas, Boryana Petrova, Naama Kanarek

Brigham and Women's Hospital, Harvard Medical School, Boston, MA 02115, USA:
Lindsey R. Baden, Kevin Mendez, Jessica Lasky-Su, Alexandra Tong, Rebecca Rooks, Michael Desjardins, Amy C. Sherman, Stephen R. Walsh, Xhoi Mitre, Jessica Cauley, Xiaofang Li, Bethany Evans, Christina Montesano, Jose Humberto Licona, Jonathan Krauss, Nicholas C. Issa, Jun Bai Park Chang, Natalie Izaguirre

Metabolon Inc, Morrisville, NC 27560, USA:
Scott R. Hutton, Greg Michelotti, Kari Wong

Prevention of Organ Failure (PROOF) Centre of Excellence, University of British Columbia, Vancouver, BC V6T 1Z3, Canada:
Scott J. Tebbutt, Casey P. Shannon

Case Western Reserve University and University Hospitals of Cleveland, Cleveland, OH 44106, USA:
Rafick-Pierre Sekaly, Slim Fourati, Grace A. McComsey, Paul Harris, Scott Sieg, George Yendewa, Mary Consolo, Heather Tribout, Susan Pereira Ribeiro

Drexel University, Tower Health Hospital, Philadelphia, PA 19104, USA:
Charles B. Cairns, Elias K. Haddad, Michele A. Kutzler, Mariana Bernui, Gina Cusimano, Jennifer Connors, Kyra Woloszczuk, David Joyner, Carolyn Edwards, Edward Lee, Edward Lin, Nataliya Melnyk, Debra L. Powell, James N. Kim, I. Michael Goonewardene, Brent Simmons, Cecilia M. Smith, Mark Martens, Brett Croen, Nicholas C. Semenza, Mathew R. Bell, Sara Furukawa, Renee McLin, George P. Tegos, Brandon Rogowski, Nathan Mege, Kristen Ulring, Pam Schearer, Judie Sheidy, Crystal Nagle

MyOwnMed Inc., Bethesda, MD 20817, USA:
Vicki Seyfert-Margolis

Emory School of Medicine, Atlanta, GA 30322, USA:
Nadine Rouphael, Steven E. Bosinger, Arun K. Boddapati, Greg K. Tharp, Kathryn L. Pellegrini, Brandi Johnson, Bernadine Panganiban, Christopher Huerta, Evan J. Anderson, Hady Samaha, Jonathan E. Sevransky, Laurel Bristow, Elizabeth Beagle, David Cowan, Sydney Hamilton, Thomas Hodder, Amer Bechnak, Andrew Cheng, Aneesh Mehta, Caroline R. Ciric, Christine Spainhour, Erin Carter, Erin M. Scherer, Jacob Usher, Kieffer Hellmeister, Laila Hussaini, Lauren Hewitt, Nina Mcnair, Susan Pereira Ribeiro, Sonia Wimalasena

Icahn School of Medicine at Mount Sinai, New York, NY 10029, USA:
Ana Fernandez-Sesma, Viviana Simon, Florian Krammer, Harm Van Bakel, Seunghee Kim-Schulze, Ana Silvia Gonzalez-Reiche, Jingjing Qi, Brian Lee, Juan Manuel Carreño, Gagandeep Singh, Ariel Raskin, Johnstone Tcheou, Zain Khalil, Adriana van de Guchte, Keith Farrugia, Zenab Khan, Geoffrey Kelly, Komal Srivastava, Lily Q. Eaker, Maria C. Bermúdez-González, Lubbertus C.F. Mulder, Katherine F. Beach, Miti Saksena, Deena Altman, Erna Kojic, Levy A. Sominsky, Arman Azad, Dominika Bielak, Hisaaki Kawabata, Temima Yellin, Miriam Fried, Leeba Sullivan, Sara Morris, Giulio Kleiner, Daniel Stadlbauer, Jayeeta Dutta, Hui Xie, Manishkumar Patel, Kai Nie, Brian Monahan

Immunai Inc., New York, NY 10016, USA:
Adeeb Rahman

Oregon Health & Science University, Portland, OR 97239, USA:
William B. Messer, Catherine L. Hough, Sarah A.R. Siegel, Peter E. Sullivan, Zhengchun Lu, Amanda E. Brunton, Matthew Strand, Zoe L. Lyski, Felicity J. Coulter, Courtney Micheletti

Stanford University School of Medicine, Palo Alto, CA 94305, USA:
Holden Maecker, Bali Pulendran, Kari C. Nadeau, Yael Rosenberg-Hasson, Michael Leipold, Natalia Sigal, Angela Rogers, Andrea Fernandes, Monali Manohar, Evan Do, Iris Chang,

Alexandra S. Lee, Catherine Blish, Henna Naz Din, Jonasel Roque, Linda N. Geng, Maja Artandi, Mark M. Davis, Neera Ahuja, Samuel S. Yang, Sharon Chinthrajah, Thomas Hagan, Tyson H. Holmes, Koji Abe

David Geffen School of Medicine at the University of California Los Angeles, Los Angeles CA 90095, USA:
Elaine F. Reed, Joanna Schaenman, Ramin Salehi-Rad, Adreanne M. Rivera, Harry C. Pickering, Subha Sen, David Elashoff, Dawn C. Ward, Jenny Brook, Estefania Ramires-Sanchez, Megan Llamas, Claudia Perdomo, Clara E. Magyar, Jennifer Fulcher

University of California San Francisco, San Francisco, CA 94115, USA:
David J. Erle, Carolyn S. Calfee, Carolyn M. Hendrickson, Kirsten N. Kangelaris, Viet Nguyen, Deanna Lee, Suzanna Chak, Rajani Ghale, Ana Gonzalez, Alejandra Jauregui, Carolyn Leroux, Luz Torres Altamirano, Ahmad Sadeed Rashid, Andrew Willmore, Prescott G. Woodruff, Matthew F. Krummel, Sidney Carrillo, Alyssa Ward, Charles R. Langelier, Ravi Patel, Michael Wilson, Ravi Dandekar, Bonny Alvarenga, Jayant Rajan, Walter Eckalbar, Andrew W. Schroeder, Gabriela K. Fragiadakis, Alexandra Tsitsiklis, Eran Mick, Yanedth Sanchez Guerrero, Christina Love, Lenka Maliskova, Michael Adkisson, Aleksandra Leligdowicz, Alexander Beagle, Arjun Rao, Austin Sigman, Bushra Samad, Cindy Curiel, Cole Shaw, Gayelan Tietje-Ulrich, Jeff Milush, Jonathan Singer, Joshua J. Vasquez, Kevin Tang, Legna Betancourt, Lekshmi Santhosh, Logan Pierce, Maria Tecero Paz, Michael Matthay, Neeta Thakur, Nicklaus Rodriguez, Nicole Sutter, Norman Jones, Pratik Sinha, Priya Prasad, Raphael Lota, Saurabh Asthana, Sharvari Bhide, Tasha Lea, Yumiko Abe-Jones

Yale School of Medicine, New Haven, CT 06510, USA:
David A. Hafler, Ruth R. Montgomery, Albert C. Shaw, Steven H. Kleinstein, Jeremy P. Gygi, Dylan Duchen, Shrikant Pawar, Anna Konstorum, Ernie Chen, Chris Cotsapas, Xiaomei Wang, Charles Dela Cruz, Akiko Iwasaki, Subhasis Mohanty, Allison Nelson, Yujiao Zhao, Shelli Farhadian, Hiromitsu Asashima, Omkar Chaudhary, Andreas Coppi, John Fournier, M. Catherine Muenker, Khadir Raddassi, Michael Rainone, William Ruff, Syim Salahuddin, Wade L. Shulz, Pavithra Vijayakumar, Haowei Wang, Esio Wunder Jr., H. Patrick Young, Albert I. Ko, Gisela Gabernet

Yale School of Public Health, New Haven, CT 06510, USA:
Denise Esserman, Leying Guan, Anderson Brito, Jessica Rothman, Nathan D. Grubaugh, Kexin Wang, Leqi Xu

Baylor College of Medicine and the Center for Translational Research on Inflammatory Diseases, Houston, TX 77030, USA:
David B. Corry, Farrah Kheradmand, Li-Zhen Song, Ebony Nelson

Oklahoma University Health Sciences Center, Oklahoma City, OK 73104, USA:
Jordan P. Metcalf, Nelson I. Agudelo Higuita, Lauren A. Sinko, J. Leland Booth, Douglas A. Drevets, Brent R. Brown

University of Arizona, Tucson AZ 85721, USA:
Monica Kraft, Chris Bime, Jarrod Mosier, Heidi Erickson, Ron Schunk, Hiroki Kimura, Michelle Conway, Dave Francisco, Allyson Molzahn, Connie Cathleen Wilson, Ron Schunk, Trina Hughes, Bianca Sierra

University of Florida, Gainesville, FL 32611, USA:
Mark A. Atkinson, Scott C. Brakenridge, Ricardo F. Ungaro, Brittany Roth Manning, Lyle Moldawer

University of Florida, Jacksonville, FL 32218, USA:
Jordan Oberhaus, Faheem W. Guirgis

University of South Florida, Tampa FL 33620, USA:
Brittney Borresen, Matthew L. Anderson

The University of Texas at Austin, Austin, TX 78712, USA:
Lauren I. R. Ehrlich, Esther Melamed, Cole Maguire, Dennis Wylie, Justin F. Rousseau, Kerin C. Hurley, Janelle N. Geltman, Nadia Siles, Jacob E. Rogers, Pablo Guaman Tipan

SUPPLEMENTAL REFERENCES

1. Ozonoff A, Jayavelu ND, Liu S, et al. Features of acute COVID-19 associated with post-acute sequelae of SARS-CoV-2 phenotypes: results from the IMPACC study. *Nat Commun*. 2024;15(1):216. doi:10.1038/s41467-023-44090-5

2. IMPACC Manuscript Writing Team, IMPACC Network Steering Committee. Immunophenotyping assessment in a COVID-19 cohort (IMPACC): A prospective longitudinal study. *Sci Immunol*. 2021;6(62):eabf3733. doi:10.1126/sciimmunol.abf3733

3. Ozonoff A, Schaenman J, Jayavelu ND, et al. Phenotypes of disease severity in a cohort of hospitalized COVID-19 patients: Results from the IMPACC study. *EBioMedicine*. 2022;83:104208. doi:10.1016/j.ebiom.2022.104208

4. Devlin NJ, Brooks R. EQ-5D and the EuroQol Group: Past, Present and Future. *Appl Health Econ Health Policy*. 2017;15(2):127-137. doi:10.1007/s40258-017-0310-5

5. Hays RD, Bjorner JB, Revicki DA, Spritzer KL, Cella D. Development of physical and mental health summary scores from the patient-reported outcomes measurement information system (PROMIS) global items. *Qual Life Res*. 2009;18(7):873-880. doi:10.1007/s11136-009-9496-9

6. Choi SW, Victorson DE, Yount S, Anton S, Cella D. Development of a conceptual framework and calibrated item banks to measure patient-reported dyspnea severity and related functional limitations. *Value Health*. 2011;14(2):291-306. doi:10.1016/j.jval.2010.06.001

7. Diray-Arce J, Fourati S, Doni Jayavelu N, et al. Multi-omic longitudinal study reveals immune correlates of clinical course among hospitalized COVID-19 patients. *Cell Reports Medicine*. 2023;4(6):101079. doi:10.1016/j.xcrm.2023.101079

8. Gygi JP, Maguire C, Patel RK, et al. Integrated longitudinal multiomics study identifies immune programs associated with acute COVID-19 severity and mortality. *J Clin Invest*. 2024;134(9). doi:10.1172/JCI176640

9. Ritchie ME, Phipson B, Wu D, et al. limma powers differential expression analyses for RNA-sequencing and microarray studies. *Nucleic Acids Research*. 2015;43(7):e47. doi:10.1093/nar/gkv007

10. Johnson WE, Li C, Rabinovic A. Adjusting batch effects in microarray expression data using empirical Bayes methods. *Biostatistics*. 2007;8(1):118-127. doi:10.1093/biostatistics/kxj037

11. Ozonoff A, Schaenman J, Jayavelu ND, et al. Phenotypes of disease severity in a cohort of hospitalized COVID-19 patients: Results from the IMPACC study. *EBioMedicine*. 2022;83:104208. doi:10.1016/j.ebiom.2022.104208

12. Amanat F, Stadlbauer D, Strohmeier S, et al. A serological assay to detect SARS-CoV-2 seroconversion in humans. *Nat Med*. 2020;26(7):1033-1036. doi:10.1038/s41591-020-0913-5

13. Argelaguet R, Velten B, Arnol D, et al. Multi-Omics Factor Analysis—a framework for unsupervised integration of multi-omics data sets. *Molecular Systems Biology*. 2018;14(6):e8124. doi:10.15252/msb.20178124

14. Gygi JP, Konstorum A, Pawar S, Aron E, Kleinstein SH, Guan L. A supervised Bayesian factor model for the identification of multi-omics signatures. *Bioinformatics*. 2024;40(5):btae202. doi:10.1093/bioinformatics/btae202

15. Bates D, Mächler M, Bolker B, Walker S. Fitting Linear Mixed-Effects Models Using lme4. *Journal of Statistical Software*. 2015;67:1-48. doi:10.18637/jss.v067.i01

16. Brysbaert M, Stevens M. Power Analysis and Effect Size in Mixed Effects Models: A Tutorial. *J Cogn*. 1(1):9. doi:10.5334/joc.10

17. Kanehisa M, Goto S. KEGG: kyoto encyclopedia of genes and genomes. *Nucleic Acids Res*. 2000;28(1):27-30. doi:10.1093/nar/28.1.27

18. Liberzon A, Birger C, Thorvaldsdóttir H, Ghandi M, Mesirov JP, Tamayo P. The Molecular Signatures Database (MSigDB) hallmark gene set collection. *Cell Syst*. 2015;1(6):417-425. doi:10.1016/j.cels.2015.12.004

19. DeHaven CD, Evans AM, Dai H, Lawton KA. Organization of GC/MS and LC/MS metabolomics data into chemical libraries. *Journal of Cheminformatics*. 2010;2(1):9. doi:10.1186/1758-2946-2-9

20. Evans AM, O'Donovan C, Playdon M, et al. Dissemination and analysis of the quality assurance (QA) and quality control (QC) practices of LC-MS based untargeted metabolomics practitioners. *Metabolomics*. 2020;16(10):113. doi:10.1007/s11306-020-01728-5

21. Evans AM, DeHaven CD, Barrett T, Mitchell M, Milgram E. Integrated, Nontargeted Ultrahigh Performance Liquid Chromatography/Electrospray Ionization Tandem Mass Spectrometry Platform for the Identification and Relative Quantification of the Small-Molecule Complement of Biological Systems. *Anal Chem*. 2009;81(16):6656-6667. doi:10.1021/ac901536h

22. Xu S, Hu E, Cai Y, et al. Using clusterProfiler to characterize multiomics data. *Nat Protoc*. 2024;19(11):3292-3320. doi:10.1038/s41596-024-01020-z

23. Liu Y, Xie J. Cauchy combination test: a powerful test with analytic p-value calculation under arbitrary dependency structures. *J Am Stat Assoc*. 2020;115(529):393-402. doi:10.1080/01621459.2018.1554485

24. Haynes WA, Vallania F, Liu C, et al. Empowering multi-cohort gene expression analysis to increase reproducibility. *Pac Symp Biocomput*. 2017;22:144-153. doi:10.1142/9789813207813_0015

25. Karisola P, Kanerva M, Vuokko A, et al. Patients with post-COVID-19 condition show minor blood transcriptomic changes, with altered erythrocyte gene expression in a male subgroup. *Front Immunol*. 2025;16:1500997. doi:10.3389/fimmu.2025.1500997

26. Hanson AL, Mulè MP, Ruffieux H, et al. Iron dysregulation and inflammatory stress erythropoiesis associates with long-term outcome of COVID-19. *Nat Immunol*. 2024;25(3):471-482. doi:10.1038/s41590-024-01754-8

27. Chen Y, Chen L, Lun ATL, Baldoni PL, Smyth GK. edgeR 4.0: powerful differential analysis of sequencing data with expanded functionality and improved support for small counts and larger datasets. Published online January 24, 2024:2024.01.21.576131. doi:10.1101/2024.01.21.576131
